# Marine Side Streams in Insect-Based Biorefineries: From Substrate–Insect Matching to Functional Aquafeed Ingredients and Bioactive Products

**DOI:** 10.3390/md24070238

**Published:** 2026-07-07

**Authors:** Beom-Seok Seo, Gahyun Kim, Hyeri Kim, Hojung Kwak, Jong-Hoon Kim

**Affiliations:** 1 Department of Biotechnology, Pukyong National University, Busan 48513, Republic of Korea; qja5106@gmail.com; 2 Department of Food Science and Technology, Pukyong National University, Busan 48513, Republic of Korea; gahyunk@pukyong.ac.kr (G.K.); hyerikim@pukyong.ac.kr (H.K.)

**Keywords:** marine by-products, insect bioconversion, aquafeed, microbiome, safety, biorefinery, bioactive compounds

## Abstract

Marine by-products, including fishery discards, seafood-processing residues, aquaculture wastes, crustacean shells, and seaweed-derived side streams, are heterogeneous feedstocks rich in proteins, lipids, minerals, chitinous materials, polysaccharides, and bioactive compounds. This review examines insect-mediated bioconversion as a controlled biorefinery strategy for transforming these unstable marine residues into functional aquafeed ingredients and value-added bioproducts. We compare major marine feedstock classes and industrially relevant insects, with emphasis on substrate–insect matching, moisture control, salinity, lipid and ash load, texture, spoilage risk, and safety. Particular attention is given to how marine substrates can tailor insect meal, insect oil, chitinous fractions, hydrolysates, frass, and functional feed additives. The review further summarizes aquafeed applications of insect-derived products, including fishmeal and fish-oil replacement, protein and amino acid quality, lipid enrichment, gut health, immunity, and disease resistance in aquatic animals. Microbiome-assisted strategies, such as fermentation, enzymatic pretreatment, and gut or substrate microbial management, are discussed as tools to improve substrate stability, digestibility, and product quality. Finally, safety, regulation, scale-up, life cycle assessment, and techno-economic issues are considered. Overall, marine insect biorefineries should be optimized not only for biomass yield, but also for product quality, traceability, and application-specific safety.

## 1. Introduction

The rapid expansion of fisheries, aquaculture, and seafood processing has intensified the need for circular strategies that can recover value from aquatic biomass beyond the edible fraction. Global aquatic food production has reached record levels, while aquaculture has become an increasingly dominant contributor to aquatic animal supply [1]. Alongside this growth, large quantities of fishery discards, seafood processing residues, aquaculture sludge, crustacean shells, mollusk residues, and seaweed-derived by-products are generated across harvesting, processing, distribution, and farming systems. These materials are often described as “waste”; however, they contain proteins, lipids, minerals, collagenous fractions, chitinous materials, pigments, polysaccharides, and other functional compounds that can support food, feed, agricultural, cosmetic, biomedical, and industrial applications [2,3]. The increasing use of processing by-products for fishmeal and fish oil production demonstrates that marine side streams are no longer merely disposal liabilities. For example, by-products accounted for a substantial share of global fishmeal and fish oil production in 2022, indicating their growing importance as secondary marine resources [1,4].

Despite their biochemical value, marine by-products are difficult to valorize in a uniform manner because they are highly heterogeneous and unstable. Fish heads, frames, viscera, skin, scales, shells, sludge, and seaweed residues differ markedly in moisture content, lipid level, ash and mineral load, salinity, texture, particle size, endogenous enzyme activity, and microbial spoilage risk. Lipid-rich fractions may undergo rapid oxidation, proteinaceous materials may produce ammonia and odor during degradation, and shellfish or sludge-derived substrates may contain high mineral contents or contaminants that require careful monitoring [2,3]. Conventional valorization routes, including collagen extraction, enzymatic hydrolysis, fish oil recovery, chitin/chitosan production, composting, silage, and anaerobic digestion, can be effective for specific and relatively clean waste streams. However, these approaches may be less suitable for mixed, wet, low-grade, or rapidly decomposing residues where standardization, transport, storage, and process economics become limiting. Therefore, complementary biological platforms capable of stabilizing heterogeneous marine residues while producing standardized outputs are needed.

Insect-mediated bioconversion has emerged as a promising approach for transforming organic residues into protein-rich biomass, functional lipids, chitin-containing fractions, frass-derived fertilizer, and other value-added materials [5,6]. Among the industrially relevant insects, the black soldier fly (BSF, *Hermetia illucens*), particularly its larval stage (BSFL), has received the greatest attention because of its rapid larval growth, broad substrate tolerance, high biomass productivity, and established relevance to animal feed and organic waste management [5,6,7]. Other insects, including mealworms, houseflies, and selected edible or feed-oriented species, may also contribute to bioconversion systems depending on substrate characteristics, regulatory context, product targets, and production infrastructure. Importantly, insects should not be viewed as replacements for all existing marine by-product valorization technologies. Rather, they can serve as biological bridging platforms for residues that are difficult to process directly through conventional extraction-based biorefineries, especially when the objective is to convert unstable, mixed, or nutrient-rich side streams into more uniform biological products.

Marine-derived substrates are particularly interesting for insect bioconversion because they can modify the nutritional and functional properties of insect biomass. Fish offal and other lipid-rich substrates have been shown to influence insect lipid accumulation and fatty acid profiles, including the potential enrichment of omega-3 fatty acids under appropriate feeding conditions [8,9,10]. Studies on mussel, fish, shrimp, seafood, and aquaculture-derived waste streams further indicate that substrate composition strongly affects larval growth, survival, conversion efficiency, biomass yield, proximate composition, and fatty acid quality [9,10,11,12,13]. These findings are highly relevant to aquafeed development because insect meals and oils are increasingly evaluated as alternatives or complements to conventional fishmeal and fish oil. In this context, marine by-products may provide not only nutrients for insect growth, but also a route to tailor insect-derived ingredients toward aquaculture-specific nutritional requirements.

However, the suitability of marine by-products for insect rearing cannot be assumed solely from their nutrient richness. High moisture content can reduce aeration and create anaerobic zones; excessive salinity or ash may impair larval performance; sharp or hard shell fragments may affect substrate structure and handling; and high lipid levels may alter microbial dynamics, oxidation, and larval fat deposition. Aquaculture sludge and seafood processing residues may also carry chemical or biological contaminants, requiring the systematic assessment of heavy metals, pathogens, antimicrobial residues, persistent organic pollutants, microplastics, and substrate-derived hazards [12,14]. These challenges highlight the need for substrate–insect matching rather than a one-size-fits-all approach. The key question is not simply whether insects can consume marine residues, but which insect species, substrate formulations, pretreatments, and process conditions can produce safe, consistent, and economically meaningful outputs.

Microbiome-assisted strategies may further expand the feasibility of marine by-product bioconversion. Insect gut microbiota and substrate-associated microorganisms can affect larval development, nutrient assimilation, microbial competition, and bioconversion performance, indicating that insect-mediated waste conversion should be understood as a host–microbe–substrate process rather than a purely insect-driven system [15,16]. Pretreatment approaches, including fermentation, enzymatic hydrolysis, bacterial inoculation, fungal pretreatment, and controlled substrate conditioning, may improve substrate palatability, nutrient availability, microbial safety, and process stability, particularly for wet, protein-rich, polymeric, or rapidly spoiling residues [15,17]. For example, protein-rich fish residues, chitin-containing crustacean shells, and polysaccharide-rich seaweed by-products may require different microbial, enzymatic, or physicochemical pretreatments to improve their compatibility with insect growth and downstream product quality. Integrating insect physiology with substrate microbiology could therefore transform marine by-products from unstable waste streams into designed feedstocks for insect-based biorefineries [16,17].

The final use of insect-derived products also requires careful consideration. In aquafeeds, insect meals are valued for their protein content, essential amino acid profile, digestibility, and potential functional effects on fish gut health, mucosal immunity, disease resistance, and stress tolerance [18,19,20]. Insect oils may provide energy and medium-chain fatty acids, whereas marine substrate enrichment can partially modify the fatty acid composition of BSFL, including increased levels of polyunsaturated fatty acids or omega-3 fatty acids under appropriate feeding conditions [21,22]. Chitin and chitosan-containing fractions from insects may support applications in feed additives, agriculture, food packaging, biomaterials, and biomedical materials, although extraction efficiency, purity, degree of deacetylation, and product standardization remain important technical considerations [23,24]. Frass and residual substrates may also contribute to nutrient recycling as organic fertilizers or soil improvers, but their agronomic use depends on nutrient composition, compost maturity, microbial safety, salinity, heavy metal accumulation, and regulatory acceptability [25,26]. Thus, the insect-based valorization of marine by-products should be understood as a multi-output biorefinery strategy rather than a single-product feed replacement approach.

**Figure 1:**
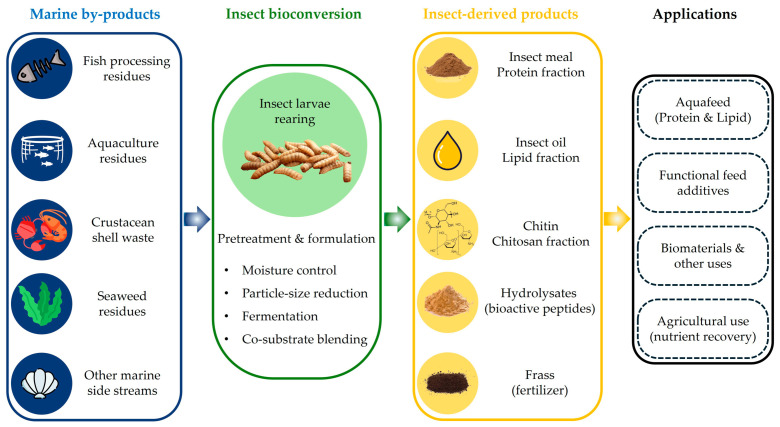
Overall framework of insect-mediated bioconversion of marine by-products into functional aquafeed ingredients and value-added bioproducts. Major marine feedstock classes include fish-processing residues, aquaculture residues, crustacean shell waste, seaweed residues, and other marine side streams. Through insect larval rearing combined with pretreatment and formulation strategies, such as moisture control, particle-size reduction, fermentation, and co-substrate blending, these heterogeneous substrates can be converted into insect meal/protein fractions, insect oil/lipid fractions, chitin- and chitosan-containing fractions, bioactive hydrolysates, and frass. These product streams can support aquafeed formulation, functional feed additives, biomaterials, and agricultural nutrient recovery.

Unlike prior reviews focused mainly on insect meal as a general fishmeal alternative or on BSFL as a broad organic-waste converter, this review specifically frames marine by-products as functional feedstocks for tunable insect-derived aquafeed ingredients, chitinous materials, lipid fractions, hydrolysates, and other marine-associated bioproducts. This review provides a comparative overview of industrially relevant insects as bioconversion platforms for fishery discards, seafood processing waste, aquaculture residues, crustacean shell waste, seaweed-derived by-products, and other marine side streams. Particular emphasis is placed on substrate–insect matching, including how moisture, texture, salinity, lipid content, mineral load, spoilage risk, and safety concerns affect insect performance and product quality. The review also discusses the potential of marine by-products to improve insect-derived aquafeed ingredients, the role of microbiome-assisted pretreatment and gut microbial functions, and the regulatory, safety, and scale-up barriers that must be addressed before marine insect biorefineries can be implemented in practical fisheries and aquaculture systems. The overall conceptual framework of insect-mediated marine by-product bioconversion, including feedstock classes, insect processing strategies, product streams, and potential applications, is summarized in Figure 1.

## 2. Marine By-Products as Feedstocks for Insect Bioconversion

Marine by-products should be considered heterogeneous biological feedstocks rather than uniform waste streams, because their suitability for insect bioconversion depends on biochemical composition, moisture, salinity, particle structure, microbial stability, and contaminant profile [2,3]. Fishery discards and seafood processing residues differ substantially according to species, anatomical fraction, habitat, season, reproductive stage, diet, freshness, and processing method [27,28]. These differences are critical for insect-based systems because substrate composition directly affects larval survival, growth rate, feed intake, conversion efficiency, biomass yield, and final larval composition [5,7]. Therefore, marine by-products should not be evaluated only by total nutrient content, but by how specific substrate traits influence host–substrate compatibility, microbial dynamics, and downstream product quality [6,15].

From an insect bioconversion perspective, marine by-products can be broadly grouped into protein-rich soft tissues, lipid-rich residues, collagenous and mineralized fractions, chitinous shell residues, polysaccharide-rich seaweed residues, and sludge-like aquaculture wastes [29,30]. Each group presents different opportunities and constraints for insect rearing [11,12]. Protein- and lipid-rich fish residues may support rapid larval growth and nutritional enrichment, whereas shell-, bone-, or sludge-rich materials may require grinding, dilution, fermentation, or co-substrate formulation before they can be used efficiently [13,15]. This section therefore reframes marine by-products as insect-rearing feedstocks and evaluates their suitability in terms of digestibility, process stability, safety, and potential to tailor insect-derived aquafeed ingredients.

### 2.1. Finfish Processing By-Products

Finfish processing generates heads, frames, backbones, skins, scales, fins, bones, viscera, liver, roe, blood, swim bladder, and trimming residues [29,31]. These fractions differ in protein, lipid, ash, collagen, mineral, and endogenous enzyme contents, which makes their value as insect substrates strongly fraction-dependent [29,32]. Heads, frames, and trimmings generally contain residual muscle, connective tissue, bone, cartilage, and lipids, whereas viscera and liver contain readily degradable proteins, digestive enzymes, bile-associated compounds, and lipid-rich fractions [31,33]. Skin, scales, fins, bones, and swim bladder are more collagenous or mineralized, which makes them less immediately digestible for insects than soft tissues but potentially useful after pretreatment or as minor components of mixed substrates [34,35].

Soft fish residues are attractive for BSFL because they provide nitrogen, essential amino acids, residual lipids, minerals, and water-soluble nutrients that can support larval growth and biomass production [8,11]. Fish offal has been directly used for BSFL recycling and was shown to produce insect biomass enriched with omega-3 fatty acids [8]. Fisheries and aquaculture by-products have also been reported to modulate BSFL growth, body composition, and omega-3 polyunsaturated fatty acid content [21]. These findings indicate that fish residues can function not only as waste-reduction substrates, but also as nutritional modulators for insect-derived aquafeed ingredients [18,21].

However, finfish residues also have several constraints as insect substrates. Viscera, blood, liver, and trimming residues are highly perishable and can rapidly undergo autolysis and microbial spoilage, which may lead to ammonia production, odor formation, pH shifts, and microbial instability [2,3]. High moisture content can reduce substrate porosity and oxygen diffusion, increasing the risk of anaerobic zones during larval rearing [5,12]. High lipid levels can alter larval fat deposition and may increase oxidation risk during substrate storage or insect biomass processing [9,36]. Bone- or scale-rich fractions can increase the ash content and reduce the digestible organic fraction available to larvae [37,38].

For these reasons, finfish by-products are likely to be most effective when used as formulated co-substrates rather than as unprocessed single substrates. Partial dewatering, homogenization, particle-size reduction, mixing with dry carbohydrate-rich materials, or controlled fermentation may improve the texture, aeration, nutrient availability, and process stability [15,22]. Substrate fermentation is particularly relevant because it can modify microbial communities and fatty acid transfer into BSFL biomass [22]. Therefore, finfish by-products are among the most promising marine substrates for insect bioconversion, but their inclusion levels and pretreatment conditions must be optimized to balance larval performance, substrate hygiene, and aquafeed-oriented nutrient enrichment [8,21].

### 2.2. Crustacean Shell Waste

Crustacean processing residues from shrimp, crab, lobster, crayfish, and krill include the heads, shells, exoskeletons, cephalothorax, tails, appendages, and hepatopancreas [30,39]. These residues contain chitin, proteins, calcium carbonate, lipids, minerals, and carotenoid pigments such as astaxanthin [30,40]. Crustacean shells are typically protein–mineral–chitin composite matrices in which chitin is tightly associated with proteins, calcium carbonate, and pigments [41,42]. This structure makes crustacean shell waste valuable for chitin, chitosan, chitooligosaccharide, pigment, and mineral recovery, but it also limits direct digestibility as an insect-rearing substrate [42,43].

For insect bioconversion, crustacean residues are more challenging than soft fish tissues because they are rigid, ash-rich, chitinous, and often saline [13,30]. Large shell particles can reduce feeding accessibility and may impair substrate handling during larval rearing [13]. Calcium carbonate and other mineral fractions can dilute digestible organic matter and increase the ash content in both the residue and the rearing system [30,41]. Residual salts from seafood processing may also impose osmotic stress on larvae if the inclusion levels are too high [2,14]. Therefore, crustacean shell waste should not be regarded as an ideal sole substrate for insect production [13,15].

Nevertheless, crustacean residues can be incorporated into insect-based systems when they are properly processed or blended. Feeding BSFL on shrimp carcasses has been proposed as a green strategy for aquaculture waste management, showing that shrimp-derived residues can be biologically converted under suitable conditions [13]. Mechanical grinding can reduce particle-size limitations and increase contact between larvae, microbes, and organic residues [15]. Fermentation or enzymatic pretreatment may partially deproteinize shell matrices, release soluble nutrients, reduce spoilage pressure, and improve substrate accessibility [44,45]. Lactic acid bacteria (LAB) and protease-producing microorganisms are particularly relevant because they can contribute to demineralization and deproteinization during crustacean-shell valorization [45,46].

In cascade biorefinery concepts, crustacean shell waste may be better positioned as a sequential feedstock rather than as a direct insect diet. High-value pigments, proteins, chitin, or mineral fractions can be recovered first, and the remaining organic residues may then be considered for insect bioconversion if their digestibility and safety are acceptable [42,44]. This approach avoids forcing insects to process highly mineralized shell matrices while still allowing residual nutrients to be recovered as insect biomass or frass [13,25]. Thus, crustacean residues are promising for integrated marine biorefineries, but their use in insect systems should rely on pretreatment, co-substrate formulation, and clear product-target definition [15,42].

### 2.3. Mollusk and Cephalopod By-Products

Mollusk and cephalopod processing generates shells, mantles, viscera, ink sacs, pens, gonadal tissues, and trimming residues [47,48]. Mollusk shells are mainly mineral-rich residues composed largely of calcium carbonate, whereas squid pens and some cephalopod structures contain β-chitin [47,48]. Soft tissues such as mantles, viscera, and trimmings contain more digestible organic matter than shells and may provide proteins, lipids, enzymes, and other bioactive compounds [47,49]. Therefore, soft mollusk and cephalopod residues are more relevant to insect feeding than mineral-dominated shells [2,3].

The main limitation of mollusk and cephalopod soft residues is their high perishability. Viscera and trimming residues can degrade rapidly and generate strong odors because of endogenous enzymes and microbial activity [2,3]. Their high moisture content may also reduce aeration and create unstable rearing conditions if they are used without drying, mixing, or fermentation [5,15]. In contrast, mineral-rich shells have low digestible organic value for larvae and are more appropriate for separate mineral recovery or as carefully controlled mineral amendments [37,47]. Therefore, these residues should be divided into soft organic fractions and hard mineral fractions before insect-based processing is considered [47,48].

Direct evidence for BSFL conversion of mollusk and cephalopod residues remains more limited than evidence for fish offal, seafood waste, aquaculture sludge, and shrimp residues [8,11,13]. Because of this evidence gap, these substrates should be discussed cautiously as potential co-substrates rather than established insect-rearing feedstocks [2,3]. Soft tissues may be useful at controlled inclusion levels with dry co-substrates, whereas shells and pens may require grinding, extraction, or microbial treatment before further biological conversion [15,41]. Future studies should compare larval performance, substrate reduction, microbial safety, and biomass composition when mollusk or cephalopod residues are included in insect-rearing diets [11,15].

### 2.4. Aquaculture Sludge and Farming-Derived Residues

Aquaculture sludge, uneaten feed, fecal solids, biofloc residues, and sedimented organic matter are generated continuously in aquaculture systems [12,14]. These materials may contain residual proteins, lipids, carbohydrates, minerals, microbial biomass, and nutrients derived from feed and fish metabolism [12]. Fresh aquaculture sludge has been evaluated as a substrate for BSFL, showing that larval treatment can contribute to sludge management and bioconversion under controlled conditions [12]. More recent work has also evaluated recirculating aquaculture system (RAS) sludge, fish trimmings, and harvest macroalgae waste blended with vegetable by-products for BSFL conversion in a larger-scale setting [50].

Despite this potential, aquaculture sludge is a higher-risk feedstock than clean seafood processing residues. Sludge may contain high ash, salts, microbial contaminants, antimicrobial residues, heavy metals, persistent organic pollutants, microplastics, or other system-dependent hazards [14]. Belghit et al. showed that BSFL reared on aquaculture sludge can accumulate nutrients but also transfer chemical and biological contaminants from sludge into larvae [14]. This distinction is critical because waste-treatment feasibility does not automatically mean that the resulting insect biomass is suitable for feed-grade use [14,18].

Aquaculture sludge also has lower and more variable digestible nutrient density than fresh fish-processing residues. A substantial fraction of the organic matter in sludge has already passed through fish digestion, microbial transformation, or sedimentation processes [12]. High water and ash contents can further reduce process efficiency and complicate larval harvesting [12,14]. Therefore, sludge-based insect bioconversion may require dewatering, co-substrate addition, contaminant screening, and post-treatment validation before inclusion in aquafeed-oriented production systems [12,15].

A realistic approach is to separate aquaculture sludge applications according to product risk level. Sludge-derived substrates may be useful for waste stabilization, frass production, or lower-risk nutrient recycling pathways if the contaminant levels are controlled [12,25]. Feed-grade insect meal from sludge-fed larvae requires stricter contaminant monitoring, pathogen control, and regulatory validation [14,18]. Blending aquaculture waste streams with vegetable by-products may improve process performance and allow for the production of tailored larval biomass, but the inclusion levels must be optimized for both larval quality and safety [50]. Thus, aquaculture sludge is promising for circular nutrient recovery but should be regarded as a regulated high-risk substrate rather than a simple feedstock.

### 2.5. Seaweed-Derived By-Products

Seaweed processing residues and seaweed-derived side streams contain polysaccharides, proteins, minerals, pigments, phenolic compounds, and variable levels of salts [2,3]. Brown, red, and green seaweeds differ in carbohydrate composition, including alginate, fucoidan, laminarin, agar, carrageenan, ulvan, cellulose, and other structural polysaccharides [51,52]. Compared with fish or shellfish residues, seaweed by-products are generally less protein-rich but can provide marine-associated nutrients, minerals, and bioactive compounds [53]. Therefore, seaweed residues are better viewed as functional co-substrates or enrichment ingredients than as primary growth substrates for most insect systems.

Seaweed-enriched BSFL media have been directly evaluated. Feeding BSFL with brown algae-enriched media increased the incorporation of eicosapentaenoic acid (EPA), iodine, and vitamin E into larval biomass [53]. However, media containing more than 50% seaweed reduced larval growth, nutrient retention, and lipid levels compared with a plant-based control medium [53]. This indicates that seaweed can nutritionally tailor BSFL biomass, but high inclusion levels can compromise larval performance [53]. Therefore, seaweed residues should be used at optimized inclusion rates rather than as sole or dominant substrates [53,54].

Seaweed species and pretreatment methods also influence BSFL fatty acid enrichment and growth performance. Subhasinghe et al. showed that seaweed combined with fish offal enhanced omega-3 levels in BSFL, and that fermentation or microwave pretreatment of *Kappaphycus alvarezii* improved larval omega-3 enrichment compared with untreated seaweed [54]. At the same time, both untreated and pretreated seaweed reduced the larval performance relative to control substrates. These results support the idea that seaweed pretreatment can improve nutritional enrichment but does not fully remove performance constraints. Therefore, seaweed-based insect substrates should be formulated with protein- or lipid-rich co-substrates such as fish offal when aquafeed-oriented fatty acid enrichment is the target.

Safety is a major issue for seaweed-derived insect substrates. Seaweeds can accumulate minerals, heavy metals, arsenic, and microbial contaminants depending on species, harvesting site, and processing conditions [55,56]. BSFL reared on seaweed-enriched media accumulated cadmium, lead, mercury, and arsenic, and higher seaweed inclusion resulted in the larval cadmium and total arsenic levels exceeding the European Union maximum limits for complete feed [56]. Low-temperature drying of seaweed can preserve nutritional quality but may increase the risk of pathogen carry-over if raw seaweed is contaminated [55]. Seaweed-fed BSFL systems therefore require washing, drying validation, microbial monitoring, contaminant screening, and inclusion-level control.

Seaweed-derived substrates may still be valuable in integrated aquaculture-waste bioconversion. Lopes et al. showed that harvest macroalgae waste, RAS sludge, and fish trimmings can be included in BSFL diets blended with vegetable by-products, generating protein-rich larval biomass under semi-large-scale conditions [50]. In that study, macroalgae inclusion was associated with lower larval crude fat content, whereas fish trimming inclusion produced larvae with higher crude fat content [50]. This suggests that seaweed and fish residues may be used strategically to tune larval biomass composition in different directions. Overall, seaweed residues are promising for marine nutrient enrichment and functional substrate design, but their high ash, salt, complex polysaccharide content, and contaminant risks make pretreatment and co-substrate formulation essential.

### 2.6. Feedstock Classification for Substrate-Insect Matching

Marine by-products can be classified into functional substrate categories for insect bioconversion: protein-rich soft tissues, lipid-rich residues, collagenous/mineralized fractions, chitinous shell residues, polysaccharide-rich seaweed residues, and sludge-like aquaculture wastes [29,30]. Protein-rich fish heads, frames, trimmings, and viscera can support larval growth but require spoilage control and moisture management [8,11]. Lipid-rich fish residues can alter insect oil composition and increase omega-3-related value, but they may also increase oxidation and handling challenges [9,21]. Chitinous crustacean residues can contribute to circular shell-waste management, but their rigid mineralized matrix usually requires grinding, pretreatment, or co-substrate blending [13,42]. Seaweed residues can enrich BSFL with marine-associated nutrients, but high inclusion levels can reduce growth and raise contaminant concerns [53,56]. Aquaculture sludge can contribute to circular nutrient recovery, but contaminant transfer and regulatory acceptability must be evaluated before feed-grade use [12,14].

This classification highlights why marine by-products cannot be regarded as interchangeable insect substrates. Successful insect bioconversion depends on matching substrate properties with insect species, larval developmental stage, moisture level, aeration, particle size, microbial status, pretreatment strategy, and final product target [5,15]. For example, BSFL may be suitable for wet and nutrient-rich mixed residues when moisture, aeration, and microbial stability are controlled [5,12]. Mealworms and other drier-substrate insects may be less compatible with wet marine residues unless drying or formulation steps are applied [18,19]. Housefly larvae and other dipteran larvae may offer additional options for protein-rich wastes, but their safety, process control, and regulatory status must be considered separately [7,18].

Therefore, the key design question is not simply whether insects can consume marine by-products. The more useful question is which insect species, substrate formulation, pretreatment method, and product pathway can convert a specific marine residue into safe and consistent biomass, oil, chitinous fractions, frass, or other bioproducts. This substrate–insect matching framework provides the basis for the next section, which compares industrially relevant insect species according to their biological traits, substrate tolerance, conversion performance, and suitability for marine by-product valorization. A substrate-oriented classification of major marine by-products, their conversion constraints, recommended insect-bioconversion strategies, and likely outputs is summarized in Table 1.

## 3. Insect Species Used for Marine By-Product Bioconversion

Marine by-products are nutritionally rich substrates, but they are not simple feedstocks for insect bioconversion. Fishery and aquaculture residues usually contain high levels of protein, lipids, moisture, minerals, and rapidly degradable nitrogenous compounds. These properties can support larval growth, but they can also cause substrate liquefaction, odor formation, ammonia accumulation, and microbial spoilage when the substrate is not properly formulated [5,57].

The suitability of insects for marine by-product conversion therefore depends strongly on species-specific feeding ecology. Dipteran larvae, especially BSFL and housefly larvae, are generally more suitable for moist and protein-rich substrates [57]. In contrast, tenebrionid larvae such as yellow mealworms and lesser mealworms are better adapted to relatively dry and particulate diets [58,59].

Among the candidate insects, BSFL have received the greatest attention for organic waste treatment. Their broad substrate tolerance and high conversion efficiency make them suitable for many organic residues, including animal-derived and aquaculture-related wastes [5,60]. Mealworms and lesser mealworms are also valuable feed insects, but their role in marine by-product valorization is more likely to involve processed or formulated marine ingredients rather than the direct treatment of wet fish waste [59,61].

### 3.1. Black Soldier Fly Larvae

BSFL are currently the most promising insect species for marine by-product bioconversion. They can rapidly convert organic residues into larval biomass rich in protein and lipids [5]. Their non-feeding adult stage also reduces some of the pest-related concerns associated with synanthropic flies [57].

Fish offal was one of the earliest marine by-products tested as a substrate for BSFL. St-Hilaire et al. showed that feeding fish offal to BSFL increased the larval lipid content and enriched the larvae with omega-3 fatty acids [8]. This finding is important because conventional BSFL biomass is usually rich in medium-chain fatty acids but relatively low in long-chain n-3 polyunsaturated fatty acids.

Aquaculture waste has also been evaluated as a BSFL substrate. Lopes et al. demonstrated that BSFL could process aquaculture-derived biowaste and convert it into larval biomass under controlled feeding conditions [62]. This supports the use of BSFL not only for food waste treatment, but also for aquaculture residue valorization.

Fisheries and aquaculture by-products can also modify larval fatty acid composition. Arena et al. reported that marine by-product-based diets altered BSFL growth, body composition, and omega-3 polyunsaturated fatty acid (PUFA) content [21]. These results suggest that marine substrates can be used for both waste reduction and the nutritional upgrading of insect biomass.

Seaweed and microalgal residues have also been explored as marine-derived feed components for BSFL. Seaweed-enriched diets can influence the mineral and nutrient composition of larvae [53]. Industrial microalgal residues can also be used to enrich BSFL biomass with residual omega-3 fatty acids [63].

However, BSFL performance is highly dependent on substrate formulation. Pure fish waste or highly lipid-rich residues may become compact, greasy, anaerobic, or difficult for larvae to penetrate. Therefore, marine by-products are often more suitable when mixed with dry or fibrous co-substrates.

Blending fish waste with fruit or vegetable residues can improve substrate structure and nutrient balance. Isibika et al. showed that the co-conversion of fish waste with fruit residues affected the BSFL conversion efficiency and larval performance [64]. This indicates that fish waste should usually be regarded as a high-protein co-substrate rather than as a stand-alone feedstock.

Overall, BSFL are best suited for the bulk treatment of fish trimmings, fish offal, aquaculture sludge, shrimp residues, and mixed seafood wastes. Their strongest advantage is the simultaneous production of insect biomass and residue reduction. Their main limitations are excessive moisture, salt, lipid load, substrate compaction, and microbial spoilage.

### 3.2. Mealworms and Lesser Mealworms

Yellow mealworms, *Tenebrio molitor* L., have strong potential as feed insects, but they are not ideal primary converters of wet marine waste. Their natural feeding ecology is more compatible with dry cereal-based or bran-based substrates [58]. Therefore, fish by-products must usually be dried, ground, fermented, hydrolyzed, or incorporated into formulated diets before being used for mealworm production.

Fish by-products can still be useful for the nutritional enrichment of mealworms. Romero-Lorente et al. tested different pretreatments of fish by-products for feeding *T. molitor* larvae [65]. They found that fish-derived diets could increase the EPA and docosahexaenoic acid (DHA) levels in larvae, but larval performance depended strongly on the physical form of the fish substrate [65].

This distinction is important for marine by-product valorization. Mealworms should not be presented as direct equivalents of BSFL. Instead, they are more suitable for upgrading processed marine ingredients into insect biomass.

For example, dried fish powder, fish hydrolysate, defatted fish meal, or finely ground seafood residues could be incorporated into dry mealworm diets. This approach may reduce spoilage risk while allowing marine nutrients to be transferred into insect biomass. However, excessive fish inclusion may reduce palatability, increase rancidity, or impair survival.

Lesser mealworms, *Alphitobius diaperinus*, share several characteristics with yellow mealworms. They can be reared on dry organic side streams and have been evaluated for circular feed production [66]. However, direct evidence for lesser mealworm conversion of marine by-products is still limited.

Several studies have shown that *A. diaperinus* performance depends strongly on diet composition and substrate quality [66,67]. These findings suggest that lesser mealworms may tolerate processed marine ingredients, but raw fish waste is unlikely to be an appropriate substrate. Therefore, lesser mealworms should currently be positioned as candidates for dry marine-ingredient incorporation rather than for wet seafood waste treatment.

### 3.3. Housefly Larvae and Other Dipteran Larvae

Housefly larvae, *Musca domestica* L., have long been studied for organic waste conversion. They grow rapidly on moist and nitrogen-rich substrates, including manure and food waste [57]. This makes them biologically relevant for marine by-product conversion.

Housefly larvae meal has also been evaluated as a feed ingredient in aquaculture. Hashizume et al. reported that processed housefly larvae preparations could replace a substantial portion of fishmeal in red seabream diets after the removal of hydrophobic fractions [68]. Wang et al. also evaluated housefly maggot meal as a fishmeal replacement in Nile tilapia diets [69].

These studies support the feed value of housefly larvae biomass. However, they do not fully establish housefly larvae as a mature platform for marine by-product treatment. Most housefly bioconversion studies still focus on manure, food waste, or mixed organic residues rather than fishery residues alone [57].

Houseflies also raise greater management concerns than BSFL. Adult houseflies are synanthropic insects and potential mechanical vectors of pathogens. Therefore, large-scale use of housefly larvae requires stricter containment, sanitation, and pathogen-control systems.

Other dipteran larvae may also be relevant because some species naturally colonize animal residues. Blowfly and flesh fly larvae can develop on meat- or fish-derived substrates. Braverman et al. reported that *Lucilia sericata* and *Sarcophaga carnaria* larvae could reduce poultry and fish waste under experimental conditions [70].

Despite this biological potential, these species are less established for feed-grade production. Their carrion-associated ecology raises concerns about pathogen transfer, odor, myiasis risk, public acceptance, and regulatory approval. For this reason, they are better described as niche candidates rather than primary industrial species.

### 3.4. Species-Specific Tolerance to Moisture, Salinity, Lipid Load, and Substrate Texture

Moisture is one of the most important factors in marine by-product bioconversion. BSFL can tolerate moist substrates better than mealworms, but excessive water can still reduce larval survival and residue separation [71]. High moisture can also create anaerobic zones and increase odor formation.

Mealworms and lesser mealworms are much less suitable for wet substrates. They perform better on dry and porous feed matrices [58,59]. Therefore, marine by-products for tenebrionid larvae should be dried, powdered, or incorporated into bran-based diets.

Salinity is another critical constraint. Marine residues may contain salt from seawater, processing brines, or salted seafood waste. Cho et al. showed that salinity reduced BSFL growth during food waste treatment [72]. Li et al. also reported that increasing food-waste salinity affected BSFL growth and biomass composition [73].

These findings are highly relevant to seafood waste. Salted fish residues, seaweed waste, and aquaculture sludge may need washing, dilution, or blending before insect treatment. This issue is especially important because the salinity tolerance data are still limited for mealworms, lesser mealworms, and housefly larvae.

Lipid load is also important. Moderate inclusion of fish-derived lipids can enrich insect biomass with valuable fatty acids [8,21]. However, excessive lipid content may reduce substrate porosity and promote rancidity. In BSFL systems, this can lower the feeding efficiency and increase process instability.

Substrate texture strongly affects larval access to nutrients. BSFL require substrates that are moist but not overly compacted. Fish paste, viscera slurry, and oily residues may require bulking agents. Shrimp shells, crab shells, and fish bones may require grinding or pretreatment before larval feeding.

For mealworms and lesser mealworms, texture is even more restrictive. These larvae require dry and particulate substrates. Therefore, raw marine by-products should not be directly supplied unless they are converted into a stable dry ingredient.

### 3.5. Comparative Performance: Survival, Larval Weight, Bioconversion Rate, Waste Reduction, Feed Conversion Ratio, Biomass Yield

Comparative performance among insect species is difficult to generalize because experimental conditions differ widely. Survival, larval weight, bioconversion rate, waste reduction, feed conversion ratio, and biomass yield are affected by larval density, feeding rate, substrate dry matter, temperature, and trial duration [5,60]. Therefore, direct numerical comparisons should be made only when studies use similar calculation methods.

Overall, BSFL currently represent the most defensible primary platform for marine by-product bioconversion because they can process moist and protein-rich residues when moisture, texture, salinity, and microbial stability are properly controlled [8,21].

Mealworms and lesser mealworms have a different role: they are better suited to dried, powdered, or formulated marine-derived ingredients rather than the direct treatment of wet seafood residues [65,66,67].

Housefly larvae and other dipterans may also convert moist animal-derived wastes, but their broader use is limited by containment, biosecurity, public acceptance, and regulatory considerations [57,70]. Thus, insect selection should be based on substrate form and product target rather than on conversion ability alone. The comparative suitability of major insect platforms for marine by-product bioconversion is summarized in Table 2, with emphasis on substrate compatibility, practical strengths, and key limitations.

## 4. Substrate–Insect Matching and Process Optimization

Marine by-products cannot be optimized for insect bioconversion using a single universal rearing strategy. Their suitability depends on the interaction between substrate composition, insect feeding ecology, larval developmental stage, pretreatment method, and final product target. Fish offal, viscera, shrimp and crab shells, seaweed residues, aquaculture sludge, and high-salt or high-moisture seafood wastes differ greatly in digestible organic matter, lipid load, mineral content, salinity, particle structure, microbial stability, and contaminant risk [2,3,14,15]. Therefore, substrate–insect matching should be regarded as a process-design framework rather than a simple substrate-screening step.

**Figure 2:**
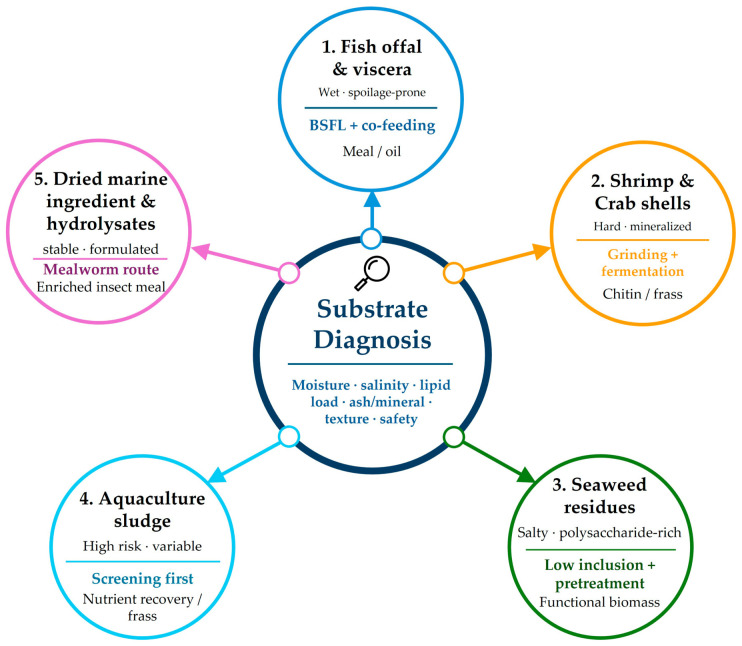
Substrate–insect matching map for marine by-product bioconversion. The central substrate-diagnosis step evaluates moisture, salinity, lipid load, ash/mineral content, texture, and safety risk before selecting an insect platform and process strategy. Wet and spoilage-prone fish offal or viscera are best directed toward BSFL-centered conversion with co-feeding, whereas mineralized shrimp and crab shells require grinding, fermentation, or other pretreatments before co-substrate use. Seaweed residues are more appropriate as low-inclusion, pretreated functional co-substrates, while aquaculture sludge should undergo safety screening before feed-grade use is considered. Dried marine ingredients and hydrolysates are more compatible with mealworm-based dry-formulation routes. BSFL, black soldier fly larvae.

In this framework, BSFL are generally positioned as the primary platform for wet, protein-rich, and mixed marine residues, whereas mealworms and lesser mealworms are more suitable for dried or formulated marine-derived ingredients [5,8,58,59]. Process optimization should then focus on adjusting the moisture, aeration, salinity, lipid load, particle size, microbial status, and co-substrate composition to improve larval survival, growth, bioconversion efficiency, biomass quality, and safety [5,15,71,74,75,76]. Based on these substrate-specific constraints, a substrate-diagnosis map can be used to guide insect species selection, pretreatment strategy, and product targeting for marine by-product bioconversion (Figure 2).

### 4.1. Fish Offal and Viscera: BSFL-Centered Conversion Strategy

Fish offal and viscera are among the most promising marine by-products for insect bioconversion because they contain digestible proteins, residual lipids, minerals, endogenous enzymes, and water-soluble nutrients [31,33]. These compositional traits are relevant to insect rearing because fish offal and fisheries- or aquaculture-derived by-products have been shown to support BSFL growth and modify larval biomass composition, including lipid and omega-3 fatty acid profiles [8,21]. Among the currently available insect platforms, BSFL are the most suitable species for these substrates because they tolerate moist and nutrient-rich organic residues better than tenebrionid larvae and have already been tested with fish offal, seafood waste, aquaculture-derived waste, and mixed fish-processing residues [8,11,12,21,62].

Fish offal feeding can also modify the larval lipid composition. St-Hilaire et al. showed that fish offal recycling by BSFL produced larvae enriched with omega-3 fatty acids, indicating that fish residues can function not only as growth substrates, but also as nutritional modulators for aquafeed-oriented insect biomass [8]. Similarly, fisheries and aquaculture by-products have been reported to alter BSFL growth, body composition, and omega-3 PUFA content [21].

However, fish offal and viscera should not be regarded as ideal sole substrates under all conditions. These materials are highly perishable and can rapidly undergo autolysis and microbial spoilage, leading to odor formation, ammonia accumulation, pH shifts, and unstable microbial dynamics [2,3,5]. Their high moisture content can reduce substrate porosity and oxygen diffusion, creating anaerobic zones that impair larval activity and complicate residue separation [5,12,71,74]. In addition, lipid-rich viscera or liver fractions may increase larval fat deposition and improve fatty acid enrichment, but excessive lipid load can reduce the substrate structure, increase rancidity risk, and destabilize the rearing bed [9,21,36].

For this reason, fish offal and viscera are best used as high-value co-substrates in BSFL-centered formulations rather than as unprocessed single feedstocks. Mixing fish residues with dry carbohydrate-rich or fibrous materials can improve the substrate texture, moisture balance, aeration, and handling [15,64,76]. Isibika et al. showed that co-conversion of fish waste with fruit residues affected BSFL conversion efficiency and larval performance, supporting the importance of substrate blending for fish-waste-based systems [64]. Practical optimization strategies may include partial dewatering, mincing or homogenization, mixing with dry plant residues, controlled fermentation, and short-term storage stabilization before larval feeding [15,22,71,74,75,76]. In aquafeed-oriented systems, the inclusion level of fish offal should be optimized not only for larval weight gain or waste reduction, but also for final insect meal quality, fatty acid profile, microbial safety, and oxidative stability.

### 4.2. Shrimp and Crab Shell Waste: Need for Co-Substrate Formulation and Pretreatment

Shrimp and crab processing residues are compositionally different from fish offal because they contain high proportions of chitin, calcium carbonate, proteins, minerals, pigments, and residual salts [30,39,40,41,42]. These properties make crustacean shell waste valuable for chitin, chitosan, pigment, and mineral recovery, but they also limit direct use as insect substrates [30,40,42]. The rigid shell matrix reduces larval access to nutrients, while high ash and calcium carbonate contents dilute digestible organic matter [30,41]. Residual salinity from seafood processing can further impose osmotic stress on larvae, especially when shell waste is used at high inclusion levels [14,72,73].

BSFL can contribute to shrimp-waste valorization under suitable conditions. Hu et al. reported that BSFL could be fed on shrimp carcasses as a green technology for aquaculture waste management and circular economy [13]. However, this does not mean that shrimp or crab shell waste should be used as a sole substrate without preparation. Crustacean shells are physically hard, mineral-rich, and poorly accessible compared with fish viscera or soft tissues. Therefore, crustacean shell waste is more defensible as a co-substrate or pretreated feedstock than as a direct insect diet [13,15].

Process optimization for shrimp and crab shell waste should prioritize particle-size reduction, demineralization, deproteinization, fermentation, and blending with digestible organic substrates. Mechanical grinding can increase the surface area and improve contact between larvae, microbes, and residual organic matter [15]. Fermentation using LAB or proteolytic microorganisms may partially solubilize proteins, reduce spoilage pressure, and improve substrate accessibility [40,45,46]. Enzymatic or microbial pretreatment may also help release soluble nutrients from the shell matrix before insect feeding [44,45]. In practical systems, shrimp or crab shell residues should be mixed with softer and more digestible substrates, such as fish offal, vegetable residues, or carbohydrate-rich by-products, to improve larval performance and reduce the negative effects of excessive ash and shell particles [13,15,76].

A cascade biorefinery approach may be more appropriate for crustacean shell waste than direct insect conversion. In this strategy, high-value compounds such as astaxanthin, proteins, chitin, or minerals can be recovered first, and the remaining organic-rich fraction can then be evaluated for insect bioconversion if its digestibility and safety are acceptable [42,44]. This approach prevents insects from being forced to process highly mineralized shell matrices while still allowing residual nutrients to be converted into insect biomass or frass. Therefore, shrimp and crab shell waste should be positioned as an integrated biorefinery feedstock requiring pretreatment and co-substrate formulation, rather than as a simple replacement for conventional BSFL diets.

### 4.3. Seaweed By-Products: Carbohydrate-Rich Substrates Requiring Microbial Pretreatment

Seaweed-derived by-products represent a distinct class of marine feedstocks because they are generally rich in structural carbohydrates, minerals, pigments, phenolic compounds, and salts, but lower in digestible protein and lipid than fish-processing residues [2,3,51,52,53]. Brown, red, and green seaweeds contain complex polysaccharides such as alginate, fucoidan, laminarin, agar, carrageenan, ulvan, cellulose, and other structural carbohydrates [51,52]. Many of these polysaccharides are not readily digestible by insects without microbial or enzymatic assistance. Therefore, seaweed residues should be considered functional co-substrates or nutrient-enrichment ingredients rather than primary growth substrates for most insect systems.

Direct studies support this cautious interpretation. Liland et al. reported that brown-algae-enriched media increased the incorporation of EPA, iodine, and vitamin E into BSFL biomass, but high seaweed inclusion reduced the larval growth, nutrient retention, and lipid content [53]. Subhasinghe et al. also showed that seaweed combined with fish offal could enhance the omega-3 levels in BSFL, and that fermentation or microwave pretreatment of *Kappaphycus alvarezii* improved larval omega-3 enrichment compared with untreated seaweed [54]. However, seaweed-containing diets still reduced the larval performance compared with control substrates, indicating that nutritional enrichment and larval productivity must be balanced [54].

The main limitation of seaweed by-products is not simply low protein content. Their high ash, salt, and complex polysaccharide contents can reduce feed efficiency, impair larval growth, and affect biomass composition [53,54]. In addition, seaweeds can accumulate iodine, arsenic, cadmium, lead, mercury, and other contaminants depending on species, cultivation area, harvest season, and processing method [55,56]. Therefore, seaweed-fed insect systems require inclusion-level control, contaminant screening, and feed-grade risk assessment.

Microbial pretreatment is particularly important for seaweed-based insect substrates. Fermentation may partially hydrolyze seaweed polysaccharides, reduce anti-nutritional or inhibitory effects, improve palatability, and alter microbial communities before larval feeding [15,54]. Enzymatic pretreatment using carbohydrate-active enzymes may also improve the release of soluble sugars from algal polysaccharides, although this strategy requires further insect-specific validation [51,52]. In practice, seaweed residues are likely to perform best when combined with protein- or lipid-rich co-substrates such as fish offal, seafood trimming residues, or plant by-products. Such formulations can use seaweed to enrich insect biomass with marine-associated micronutrients while maintaining sufficient digestible nutrients for larval growth [50,53,54].

### 4.4. Aquaculture Sludge: High Circular-Economy Potential but Major Safety and Regulatory Risk

Aquaculture sludge, uneaten feed, fecal solids, biofloc residues, and sedimented organic matter are attractive from a circular-economy perspective because they represent continuous nutrient losses from aquaculture production systems [12,14]. These materials may contain residual proteins, lipids, carbohydrates, minerals, microbial biomass, and feed-derived nutrients [12]. BSFL have been evaluated for fresh aquaculture sludge management, and larval treatment can contribute to sludge reduction and nutrient recovery under controlled conditions [12]. Larger-scale work has also tested RAS sludge, fish trimmings, harvest macroalgae waste, and vegetable by-products in BSFL diets, showing that aquaculture-related residues can be incorporated into insect-rearing systems when properly formulated [50].

Nevertheless, aquaculture sludge should be regarded as a high-risk substrate rather than a simple feedstock. Unlike clean fish-processing by-products, sludge may contain fecal matter, microbial contaminants, high ash, salts, antimicrobial residues, heavy metals, persistent organic pollutants, microplastics, and other system-dependent hazards [14]. Belghit et al. demonstrated that BSFL reared on aquaculture sludge can accumulate nutrients but may also transfer chemical and biological contaminants from sludge into larvae [14]. This is a critical point because successful larval growth does not automatically mean that the resulting biomass is suitable for aquafeed use.

From a process-optimization perspective, aquaculture sludge requires dewatering, homogenization, contaminant screening, pathogen control, and co-substrate blending [12,14,15]. Dewatering is necessary because sludge often has a low dry matter content, which reduces process efficiency and complicates larval harvesting [12,74]. Co-substrate addition can improve digestible nutrient density, substrate structure, and larval performance [50,76]. However, even when larval growth is acceptable, feed-grade application requires the strict validation of heavy metals, pathogens, antimicrobial residues, microplastics, and other contaminants [14,18].

Therefore, the most realistic strategy is to separate sludge-based applications according to product risk level. If the target is waste stabilization, nutrient recovery, or frass production, aquaculture sludge may be useful under controlled safety conditions [12,25]. If the target is feed-grade insect meal or oil for aquaculture, the regulatory burden becomes much higher, and sludge inclusion should be limited or avoided unless contaminant transfer is thoroughly assessed [14,18]. In this context, aquaculture sludge is promising for circular nutrient recovery, but it is less defensible than fish offal or clean processing residues for the direct production of aquafeed-grade insect ingredients.

### 4.5. Managing High-Salt and High-Moisture Substrates: Dilution, Drying, Co-Feeding, and Fermentation

High salinity and high moisture are two of the most important process constraints in marine by-product bioconversion. Seafood residues may contain salt from seawater, processing brines, salted products, seaweed tissues, or aquaculture sludge [14,55,56]. Salinity can reduce larval growth, alter biomass composition, and impose osmotic stress. Cho et al. showed that salinity reduced BSFL growth during food waste treatment, while Li et al. reported that increasing salinity affected BSFL growth and biomass composition [72,73]. These findings are directly relevant to salted seafood residues, seaweed waste, and aquaculture sludge. Therefore, salinity should be measured and controlled before marine by-products are introduced into insect-rearing systems.

Dilution and co-feeding are the simplest approaches for managing salinity. High-salt substrates can be blended with low-salt dry materials, vegetable by-products, cereal bran, fruit residues, or other organic side streams to reduce osmotic stress and improve substrate structure [15,64,76]. Washing or soaking may reduce surface salts in seaweed or shell residues, but this creates wastewater that must be managed. Therefore, salt-reduction strategies should be evaluated not only by larval performance but also by water use, effluent generation, cost, and environmental trade-offs.

Moisture optimization is equally important. BSFL tolerate moist substrates better than mealworms or lesser mealworms, but excessive moisture can cause substrate compaction, oxygen limitation, anaerobic zones, odor generation, poor residue separation, and low process efficiency [5,71,74]. Cheng et al. showed that residue separation by sieving was feasible when food waste had 70–75% moisture, whereas 80% moisture made residue sieving difficult [71]. Lalander et al. further demonstrated that very high-water-content substrates reduced BSFL biomass conversion and survival, showing that water content must be regarded as a core process variable rather than a minor formulation issue [74].

The optimal moisture range may also depend on substrate type, reactor design, aeration, larval density, and desired output. Bekker et al. showed that substrate moisture content influenced BSFL growth and metabolic performance, supporting the need to optimize moisture according to rearing conditions [75]. For marine by-products, this is especially relevant because fish viscera, seafood-processing slurries, and aquaculture sludge often contain excessive water and can rapidly become compacted or anaerobic [12,14]. Therefore, wet marine substrates often require drying, dewatering, bulking agents, or absorbent co-substrates before larval feeding [12,15,74,76].

Dry material addition is a practical moisture-control strategy. Laksanawimol et al. evaluated different dry materials for controlling moisture in BSFL rearing substrates, supporting the use of dry ingredients as formulation tools for high-moisture feedstocks [76]. In marine by-product systems, candidate bulking or absorbent materials may include cereal bran, rice bran, spent grain, fruit pomace, vegetable residues, sawdust-like lignocellulosic materials, or other low-salt dry side streams, depending on local availability and feed-safety requirements. However, these materials should not be selected only for water absorption. Their digestible nutrient content, fiber level, microbial load, cost, and effect on final larval composition should also be considered [15,64,76].

Fermentation can be used as a multifunctional pretreatment for high-moisture marine substrates. Controlled fermentation may stabilize rapidly spoiling substrates, modify microbial communities, improve palatability, reduce pathogen pressure, and alter fatty acid transfer into BSFL biomass [15,22,54]. Substrate fermentation has also been shown to modulate BSFL fatty acid composition, suggesting that microbial conditioning can be used not only for process stabilization but also for product-quality design [22]. However, fermentation must be carefully controlled because uncontrolled microbial activity can increase ammonia, organic acid accumulation, odor, or nutrient loss [5,15].

Overall, process optimization should follow a decision-tree logic. The substrate should first be classified according to its dominant limitation, such as excessive moisture, high salinity, lipid load, hard shell texture, complex polysaccharides, or contaminant risk. The insect platform should then be selected accordingly: BSFL for wet and mixed marine residues, and mealworms or lesser mealworms for dry formulated ingredients [5,58,59]. Finally, pretreatment and formulation should be matched with the intended output, whether feed-grade insect meal, insect oil, chitinous fractions, frass, or waste-stabilization products. This shifts marine insect bioconversion from opportunistic waste feeding toward controlled feedstock engineering. Representative studies demonstrating how different marine by-products, insect platforms, and pretreatment or co-feeding strategies influence bioconversion performance and product quality are summarized in Table 3.

When interpreting the studies summarized in Table 3, the evidence should be distinguished by type. Direct conversion evidence refers to studies in which insects were reared on fish offal, seafood waste, aquaculture sludge, seaweed, shell waste, or related marine residues. Substrate-enrichment evidence refers to studies where marine-derived ingredients were added to insect diets to modify biomass composition. Aquafeed application evidence refers to feeding trials using insect meal or oil, which may not always involve marine-fed insects. Extrapolated or process evidence includes microbiome, pretreatment, safety, life cycle, techno-economic, and regulatory studies that support framework development but require further validation in marine by-product systems.

## 5. Microbiome-Assisted Insect Bioconversion

**Figure 3:**
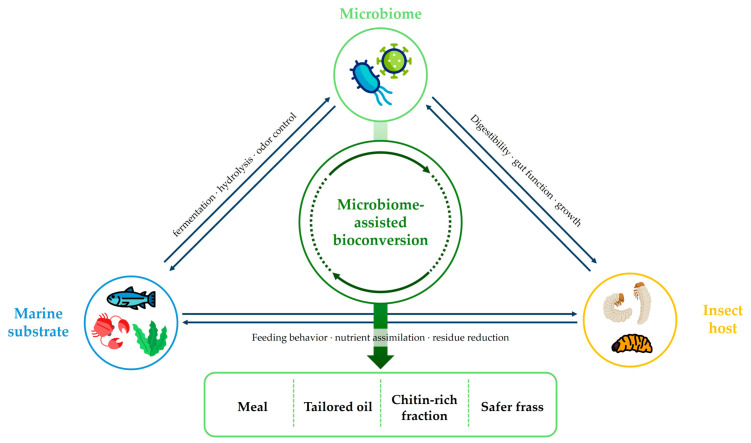
Host–microbe–substrate interaction model for microbiome-assisted insect bioconversion of marine by-products. Marine substrates, insect hosts, and substrate- or gut-associated microbiomes interact bidirectionally during insect-mediated bioconversion. Microbial fermentation, hydrolysis, odor control, and substrate stabilization can improve the accessibility of marine residues, while the insect host influences feeding behavior, nutrient assimilation, gut function, larval growth, and residue reduction. Managing these interactions can support the production of insect meal, tailored insect oil, chitin-rich fractions, and safer frass from heterogeneous marine by-products.

Insect-based bioconversion should be understood as a host–microbiome–substrate process rather than a simple larval feeding system. The gut microbiota of waste-converting insects can contribute to digestion, enzyme production, nutrient release, pathogen suppression, and adaptation to heterogeneous organic substrates [16,77,78]. This concept is particularly important for marine by-products because fish offal, viscera, crustacean shells, seaweed residues, and aquaculture-derived wastes differ markedly in moisture, salinity, lipid content, protein composition, mineral load, and microbial spoilage risk [79,80,81,82,83]. Therefore, microbiome-assisted insect bioconversion provides a rational strategy to improve substrate stabilization, nutrient accessibility, odor control, and conversion efficiency. This host–microbiome–substrate interaction model provides the conceptual basis for microbiome-assisted marine by-product bioconversion (Figure 3).

Among the waste-converting insects, BSFL remain the most developed model for microbiome-assisted bioconversion. Recent studies indicate that the BSFL gut microbiome is not merely a passive reflection of the diet, but may participate in the degradation of complex biological side streams and support larval adaptation to variable substrates [77,84,85]. For example, metagenomic and functional analyses have shown that BSFL-associated microbiota are linked to the biodegradation of industrial side streams and the enrichment of carbohydrate-, protein-, and lipid-processing functions [84]. Moreover, BSFL may recruit functional microbes from the surrounding substrate environment, suggesting that larval performance can be influenced by both gut-resident and substrate-derived microbial communities [85]. This is directly relevant to marine residues, where substrate-associated microorganisms can rapidly shift toward spoilage, fermentation, or stabilization depending on process conditions.

For fish offal and viscera, microbial conditioning is especially important because these substrates are protein- and lipid-rich but highly susceptible to putrefaction, ammonia generation, lipid oxidation, and off-odor formation. LAB-based fermentation has been used to valorize fish by-products and acid whey into bioactive fish protein hydrolysates, supporting the idea that controlled fermentation can convert unstable fish residues into more predictable protein-rich substrates [79]. Solid-state fermentation with probiotic strains has also been reported to improve the nutritional value of fish waste, indicating that microbial pretreatment can function as a value-enhancing step rather than merely a preservation method [80]. In this context, LAB fermentation before larval inoculation may reduce uncontrolled spoilage and partially hydrolyze proteins, thereby improving compatibility between fish-derived substrates and insect digestion.

Crustacean shell residues require a different microbiome-assisted strategy. Shrimp and crab shells are structurally resistant because chitin is embedded within protein–mineral matrices. Direct larval feeding on untreated shell waste is therefore likely to be inefficient unless the shell matrix is mechanically reduced, fermented, enzymatically treated, or mixed with digestible co-substrates. Recent studies on shrimp-shell bioprocessing show that microbial fermentation can promote deproteinization and demineralization, improving chitin recovery and substrate accessibility [81,82,83]. Co-fermentation systems using proteolytic and lactic acid-producing microorganisms have achieved high deproteinization and demineralization efficiencies, supporting their use as pretreatment tools before insect-based conversion [82,83]. For marine insect bioconversion, this means that shrimp and crab shells should not be regarded as simple feed ingredients, but as chitinous substrates requiring microbial opening of the matrix.

Seaweed residues require yet another microbial strategy because their major bottleneck is polysaccharide accessibility rather than protein or lipid degradation. Brown seaweeds contain alginate, laminarin, fucoidan, cellulose, and other carbohydrate fractions, while red and green seaweeds contain different sulfated and structural polysaccharides [51,52,86]. Marine bacteria capable of degrading alginate and laminarin have been proposed for the reutilization of brown-seaweed waste, indicating that seaweed valorization often depends on carbohydrate-active microbial functions [86]. Therefore, seaweed–insect bioconversion should be framed as a co-feeding and microbial-pretreatment system rather than a direct feeding process. In practical terms, seaweed residues may require microbial hydrolysis, dilution with protein-rich substrates, or fermentation with carbohydrate-active consortia before they can be efficiently incorporated into insect rearing systems.

Although BSFL are the most established model, microbiome-assisted bioconversion should also be extended to other insect platforms with distinct substrate preferences. Yellow mealworm larvae (*Tenebrio molitor*) also have a gut microbiome that responds to diet shifts and participates in the utilization of complex substrates [87,88]. Studies using polystyrene, polyethylene, and lignocellulosic materials have shown that mealworm gut microbial communities change according to substrate type, implying that the mealworm gut environment can support microbial adaptation to recalcitrant diets [87,88]. Probiotic supplementation studies in *T. molitor* further suggest that microbial manipulation is possible, although the effects on larval performance may be substrate- and strain-dependent [89]. For marine by-products, *T. molitor* should therefore be positioned not as a primary species for wet fish offal, but as a candidate for dry or semi-dry formulations containing dried fish residues, defatted fish meal fractions, shrimp-shell powder, crab-shell powder, or seaweed-containing composite diets.

The lesser mealworm (*Alphitobius diaperinus*) should be discussed more cautiously because its direct application to marine by-product bioconversion remains limited. Nevertheless, recent work on *A. diaperinus* has examined its ability to process synthetic plastic wastes and has highlighted the potential involvement of gut-associated microorganisms in difficult-substrate utilization [90,91]. In addition, studies on culturable gut bacteria from *A. diaperinus* larvae suggest that this insect harbors microbial taxa that may be relevant to substrate transformation, although their functional roles remain insufficiently characterized [91]. Therefore, the lesser mealworm should be described as a promising but underexplored candidate for dry, formulated, or chitin-supplemented marine by-product diets rather than as a proven organism for wet seafood waste treatment.

Housefly larvae (*Musca domestica*) provide another important microbiome-assisted dipteran model. The gut microbiota of housefly larvae is often dominated by Proteobacteria and Firmicutes, and feeding activity can transfer gut-associated microorganisms back into the substrate, potentially affecting substrate microbial succession [92]. More recent work showed that functional gut microbiomes can enhance housefly larval performance on waste- and by-product-based substrates, supporting the idea that fly-larval bioconversion can be optimized through microbial management [93]. Industrial-scale studies also indicate that the gut microbiota of *M. domestica* larvae changes dynamically during organic residue conversion [94]. Therefore, housefly larvae may provide a useful comparative model for high-moisture marine residues where rapid microbial turnover, pathogen monitoring, and substrate stabilization are major concerns.

Microbial starters can be applied at several stages of marine by-product insect bioconversion. First, substrate-level pretreatment can be performed before larval inoculation using LAB, *Bacillus*, yeasts, fungi, or marine polysaccharide-degrading bacteria [79,80,81,82,83,86]. LAB are most relevant for acidification and spoilage control in fish-derived substrates [79,80]. Proteolytic and chitinolytic bacteria are particularly relevant for crustacean shells because deproteinization and chitin accessibility are central bottlenecks [81,82,83]. Carbohydrate-active marine bacteria are more appropriate for seaweed residues, where alginate, laminarin, and other marine polysaccharides limit digestibility [86,95]. Second, co-feeding can be used to balance substrate moisture, salt, carbon-to-nitrogen ratio, and texture. Third, insect gut microbiome modulation may be attempted through microbial supplementation, although stable colonization should not be assumed because gut communities are strongly shaped by diet, developmental stage, and rearing environment [77,84,85,89,93].

The key microbial functions required for marine by-product bioconversion include proteolysis, lipolysis, chitinolysis, polysaccharide degradation, antimicrobial activity, and odor suppression. Proteolytic microbes can convert fish muscle residues, viscera proteins, and collagen-rich fractions into peptides and amino acids [79,80]. Lipolytic microbes may help transform fish oil and viscera-derived lipids, potentially affecting larval fat accumulation and fatty acid composition. Chitinolytic and proteolytic bacteria can loosen shrimp and crab shell matrices by degrading associated proteins and chitin-containing structures [81,82,83]. Marine polysaccharide-degrading microbes can improve the accessibility of seaweed carbohydrates, especially alginate and laminarin [86]. Thus, microbiome-assisted insect bioconversion should be designed as a substrate-specific microbial matching strategy rather than a generic probiotic addition.

Safety control is also essential because microbiome-assisted systems deliberately manipulate microbial communities. Marine by-products may contain spoilage bacteria, histamine-producing microorganisms, pathogens, excess salt, oxidized lipids, and heavy metals. Insect larvae and their residues may also contain substrate-derived or gut-associated bacteria that vary according to rearing conditions [77,92,93,94]. Therefore, starter strains should be selected based on genome-level safety, absence of virulence genes, absence of transferable antimicrobial-resistance determinants, enzyme profiles, salt tolerance, compatibility with the target insect species, and regulatory acceptability. Process monitoring should include pH, moisture, ammonia, volatile compounds, pathogen indicators, larval survival, biomass yield, waste reduction, and final product composition.

Overall, microbiome-assisted insect bioconversion provides a strong framework for upgrading marine by-products that are difficult to process through insect feeding alone. BSFL remain the most practical model for wet, nutrient-rich, and rapidly degradable marine residues, but mealworms, lesser mealworms, and housefly larvae should be included as complementary systems with distinct substrate preferences and microbiome functions. By combining insect species selection with substrate-specific microbial pretreatment, co-feeding, and safety monitoring, marine by-product bioconversion can be developed into a more controlled, scalable, and biologically optimized valorization platform.

## 6. Insect-Derived Products from Marine By-Product Feeding

Insect-mediated marine by-product valorization should not be evaluated only by larval growth or waste reduction. Its practical value depends on the quality, safety, and functionality of the products generated after feeding insects with marine-derived substrates. In this context, marine by-products can be used not only as low-cost rearing substrates, but also as nutritional modulators that reshape insect meal, insect oil, chitinous fractions, frass, and other functional outputs [8,18,21,22,53,54]. This product-oriented perspective is important because the same substrate may be suitable for one output but unsuitable for another. For example, fish offal may improve larval lipid deposition and omega-3 fatty acid enrichment, whereas aquaculture sludge may contribute to nutrient recovery but raise stronger concerns regarding contaminant transfer into feed-grade biomass [8,14,21]. Therefore, insect-derived products from marine by-product feeding should be assessed according to their intended use, including aquafeed ingredients, lipid sources, chitin/chitosan materials, organic fertilizers, or functional feed additives.

### 6.1. Insect Meal: Protein Quality, Amino Acids, and Digestibility

Insect meal is the primary product of most insect-based bioconversion systems and is particularly relevant for aquafeed formulation. BSFL meal, mealworm meal, lesser mealworm meal, and housefly larvae meal have been evaluated as alternative or complementary protein sources for fish and crustacean feeds [18,19,57,68,69]. Their value depends not only on the crude protein content, but also on the essential amino acid balance, protein digestibility, lipid level, ash content, chitin content, and processing method [18,19,96,97]. This distinction is important because the crude protein values in insect meals may be overestimated when non-protein nitrogen from chitin or other nitrogenous compounds are included in conventional nitrogen-to-protein conversion calculations [18,23].

Marine by-product feeding can modify the nutritional value of insect meal in several ways. Protein-rich fish offal, trimmings, viscera, and aquaculture by-products can increase the availability of nitrogenous nutrients and may support the production of larvae with favorable amino acid profiles for aquafeed use [8,21,62]. Fish-derived substrates may also supply minerals and marine-associated nutrients that are less abundant in plant-based rearing substrates [21,50]. However, high ash, salt, or mineral-rich fractions such as fish bones, shells, seaweed residues, and aquaculture sludge may increase the ash content of the final insect meal and reduce the proportion of digestible organic matter [14,30,56]. Thus, a substrate that improves biomass production does not necessarily produce an optimal aquafeed ingredient.

Digestibility is a central quality criterion for insect meal. In Atlantic salmon, BSFL meal has been reported to show high protein digestibility and acceptable amino acid digestibility, supporting its use as a fish feed ingredient [96,97]. However, digestibility can vary according to insect species, larval stage, substrate composition, defatting, drying temperature, particle size, and chitin content [18,19,97]. Marine-fed insect meals therefore require product-specific evaluation rather than simple extrapolation from conventional grain- or food-waste-fed insects. For example, fish-offal-fed BSFL meal may have improved lipid and mineral composition, whereas shrimp-shell- or seaweed-supplemented larvae may contain higher ash, chitin, iodine, or contaminant levels [53,56]. Therefore, proximate composition, amino acid profile, apparent digestibility coefficients, mineral balance, and contaminant levels should be measured together when marine-fed insect meal is intended for aquafeed use.

Processing also determines the final value of insect meal. Defatting can concentrate protein and improve formulation flexibility, but it changes the energy content and may remove lipid-associated functional compounds [18,19]. Heat treatment improves microbial safety but can reduce amino acid availability if excessive Maillard reactions occur, especially in substrates rich in proteins and reducing sugars [18,97]. Enzymatic hydrolysis or fermentation may improve protein solubility and peptide release, creating hydrolyzed insect protein ingredients with potential functional value [15,97]. Thus, marine-fed insect meal should be considered not as a single ingredient category, but as a family of products shaped by substrate, insect species, and downstream processing. Although several aquafeed studies have reported the amino acid composition and apparent amino acid digestibility of insect meals, direct optimization of essential amino acid profiles through controlled marine by-product formulation remains limited. Therefore, future studies should optimize substrate blends not only for larval growth or biomass yield, but also for target-species-specific amino acid balance, digestible protein supply, ash and chitin levels, palatability, and feed-chain safety.

### 6.2. Insect Oil: Lauric Acid, Polyunsaturated Fatty Acid Enrichment, and Marine Fatty Acid Tailoring

Insect oil is another major product of insect bioconversion, especially in BSFL systems. Conventional BSFL oil is generally characterized by high levels of saturated and medium-chain fatty acids, particularly lauric acid, which can provide energy and may contribute to antimicrobial or gut-modulating effects in aquafeeds [18,19,98]. However, compared with fish oil, standard BSFL oil is usually limited in long-chain omega-3 polyunsaturated fatty acids, especially EPA and DHA [8,21,53,54]. This is a critical limitation for marine aquafeeds because EPA and DHA are essential for fish physiology, product quality, and the nutritional value of farmed seafood.

Marine by-products provide a logical strategy to tailor insect oil composition. Fish offal feeding was shown to enrich BSFL with omega-3 fatty acids, demonstrating that substrate-derived marine lipids can be transferred into larval biomass [8]. Fisheries and aquaculture by-products also modulated BSFL body composition and omega-3 PUFA content [21]. In addition, industrial *Schizochytrium* microalgal waste and seaweed-containing diets have been used to increase residual omega-3 fatty acids or marine-associated nutrients in BSFL biomass [53,63]. These studies support the concept that marine substrates can shift insect oil from a primarily terrestrial-like saturated lipid source toward a more aquafeed-relevant lipid ingredient.

Nevertheless, EPA/DHA enrichment should not be overclaimed. The fatty acid profile of BSFL reflects both dietary fatty acid transfer and insect lipid metabolism, and high marine substrate inclusion can reduce larval growth or introduce safety concerns [21,53,54,56]. Seaweed-enriched media increased the EPA, iodine, and vitamin E incorporation into BSFL biomass, but high seaweed inclusion reduced larval growth and lipid retention [53]. Fermented or pretreated seaweed combined with fish offal improved omega-3 enrichment, but larval performance remained lower than in control substrates [54]. Therefore, marine lipid enrichment must balance fatty acid quality against larval productivity, oxidative stability, cost, and contaminant risk.

For aquafeed formulation, insect oil should be positioned as a partial lipid source rather than a full replacement for marine fish oil. BSFL oil can replace vegetable oil or partially replace fish oil in some aquafeed contexts without impairing growth at moderate inclusion levels [98,99]. However, because BSFL oil is generally low in EPA and DHA unless specifically enriched, it may need to be blended with fish oil, algal oil, or marine-enriched insect oil to meet species-specific essential fatty acid requirements [8,21,53,54]. Marine by-product-fed insects therefore offer a promising route for producing tailored lipid ingredients, but fatty acid profiling, peroxide value, anisidine value, thiobarbituric acid-reactive substances (TBARS), and storage stability should be included as routine quality parameters.

### 6.3. Insect Chitin and Chitosan: Comparison with Marine Chitin

Chitin and chitosan-containing fractions represent an important non-protein product stream from insect bioconversion. Insects contain chitin in their exoskeleton, molts, and structural cuticular tissues, whereas marine chitin is commonly obtained from crustacean shells, squid pens, and other chitinous marine residues [23,24,41,47,48]. The comparison between insect chitin and marine chitin is important in this review because marine by-products may provide both the substrate and the benchmark material against which insect-derived chitin is evaluated.

Marine crustacean shells are typically composed of chitin, proteins, calcium carbonate, minerals, and pigments, which require deproteinization, demineralization, and depigmentation during chitin extraction [30,41,42]. Insect chitin is also associated with proteins, lipids, minerals, and pigments, but it generally originates from a different biological matrix and may require different extraction conditions [23,24]. Compared with crustacean shell waste, insect biomass may offer a more controllable and standardized chitin source if rearing conditions, developmental stage, and downstream processing are properly managed [23,24]. However, insect-derived chitin is not automatically superior. Extraction yield, purity, molecular weight, degree of acetylation, degree of deacetylation, viscosity, ash content, and residual protein must be standardized before it can be used in feed, packaging, biomedical, or agricultural applications [23,24].

Marine by-product feeding may influence the quality of insect-derived chitin indirectly. Chitin itself is a structural polymer and may not be fundamentally changed by diet, but the surrounding matrix can be altered by substrate-derived minerals, salts, pigments, and contaminants. Feeding larvae with shrimp-shell powder or crab-shell residues could increase the amount of chitinous or mineralized material in the rearing system, but this does not necessarily mean that the larval chitin quality improves [13,30,42]. Similarly, seaweed- or sludge-derived substrates may increase the mineral or heavy-metal burdens in larval biomass, which would complicate chitin purification [14,56]. Therefore, marine-fed insect chitin should be assessed using extraction yield, residual ash, residual protein, heavy metals, microbial safety, and physicochemical properties rather than only the total chitin content.

In aquafeeds, chitin and chitosan can act as functional components, but their effects are dose-, species-, and processing-dependent. Recent reviews indicate that dietary chitin can modulate gut microbiota and immune responses in aquatic organisms, but excessive chitin inclusion may reduce protein and lipid digestibility [100]. This dual effect is especially relevant for insect meals, because chitin can be both a functional compound and an anti-nutritional factor depending on inclusion level and target species [18,20,100]. Thus, marine-fed insect products should distinguish between whole insect meal containing native chitin, de-chitinized insect protein meal, purified insect chitin, and insect-derived chitosan. Each product has different nutritional and functional implications.

### 6.4. Frass and Residual Substrate: Fertilizer Value and Marine-Specific Risks

Frass is a composite residual product consisting of insect feces, undigested substrate, exuviae, microbial biomass, and transformed organic matter [25,26,101]. It is increasingly considered as an organic fertilizer or soil amendment because it can contain nitrogen, phosphorus, potassium, micronutrients, organic matter, chitinous residues, humic-like substances, and plant-growth-associated microorganisms [25,26,101]. In marine by-product bioconversion, frass represents an important route for nutrient recycling because not all nitrogen, minerals, and organic matter are converted into insect biomass.

Marine-fed frass may have specific agronomic advantages. Fish offal, seafood residues, seaweed by-products, and aquaculture sludge can supply nitrogen, phosphorus, calcium, magnesium, sulfur, iodine, and trace minerals that may enrich the residual fertilizer fraction [12,50,53,56]. Chitinous residues from shrimp shells, crab shells, or insect molts may also contribute to soil microbial stimulation or plant defense-related effects [25,26]. Therefore, frass from marine by-product feeding could be positioned as a nutrient-rich soil amendment within a circular blue-bioeconomy system.

However, marine-fed frass also carries specific risks that are stronger than those of conventional plant-waste-derived frass. Salinity is a major issue because seafood residues, seaweed, and aquaculture sludge can contain high salt levels [55,56,72,73]. High electrical conductivity may impair seed germination, plant growth, and soil structure if frass is applied without dilution or composting. Heavy metals and metalloids are another concern, especially when seaweed, shellfish residues, or aquaculture sludge are used [14,56]. Biancarosa et al. showed that seaweed-fed BSFL can accumulate cadmium, lead, mercury, and arsenic, indicating that both larval biomass and residual fractions require contaminant monitoring [56]. Pathogens, antimicrobial residues, microplastics, and organic pollutants may also be relevant depending on the marine substrate source [14].

Therefore, frass from marine by-product-fed insects should not be promoted simply as fertilizer without quality control. Minimum evaluation should include pH, electrical conductivity, moisture, organic matter, total nitrogen, ammonium, nitrate, phosphorus, potassium, C/N ratio, maturity index, phytotoxicity, heavy metals, arsenic species where relevant, pathogen indicators, and residual salt [25,26,56,101]. Composting or post-treatment may be necessary to improve maturity, reduce microbial risks, stabilize nitrogen, and lower phytotoxicity [25,26]. In this sense, frass is not merely a waste residue from insect production, but a secondary product whose value depends strongly on marine substrate safety and post-processing.

### 6.5. Bioactive and Functional Effects in Aquafeed

Marine-fed insect products may provide functional benefits beyond basic nutrient replacement. Insect meals can contain antimicrobial peptides, chitin, chitosan-like compounds, medium-chain fatty acids, bioactive peptides, minerals, and microbial metabolites that may influence gut health, mucosal immunity, oxidative status, stress response, and disease resistance in fish [18,19,20,100]. Recent work under farm-like conditions showed that BSFL meal can modulate immune-related parameters in Atlantic salmon, supporting the idea that insect meal may function as more than a protein substitute [100]. However, functional effects are often species-specific and depend on inclusion level, processing, diet formulation, and the health status of the target animal [18,19,20].

Marine by-product feeding could strengthen this functional-feed concept by enriching insect products with marine-associated nutrients. Fish-offal-fed larvae may contain more omega-3 fatty acids, while seaweed- or microalgal-residue-fed larvae may provide marine micronutrients, vitamin E, iodine, or residual long-chain PUFA [8,53,54,63]. These enriched products may be especially relevant for aquafeeds because they partially align insect-derived ingredients with the nutritional requirements of aquatic animals. However, enrichment must be separated from safety. The same seaweed substrate that increases iodine or EPA may also increase arsenic or cadmium risk [53,56]. Similarly, aquaculture sludge may recover nutrients but may not be appropriate for feed-grade insect biomass unless contaminant transfer is strictly controlled [14].

A useful way to frame marine-fed insect products is to divide them into three functional categories. First, nutritional ingredients include insect meal and insect oil used to replace or complement fishmeal, soybean meal, vegetable oils, or fish oil [18,19,96,98,99]. Second, functional feed additives include partially hydrolyzed insect proteins, chitin/chitosan fractions, antimicrobial peptide-rich extracts, or lauric-acid-rich lipid fractions [20,23,24,100]. Third, circular nutrient products include frass and residual substrates used for soil amendment or fertilizer applications [25,26,101]. This classification avoids treating insect biomass as a single generic ingredient and instead links each product to its most defensible application.

Overall, marine by-product feeding can transform insects into tunable bioproduct platforms. Fish residues are most promising for protein-rich biomass and lipid enrichment; seaweed and microalgal residues are promising for marine micronutrient and PUFA tailoring but require strict contaminant control; crustacean shell residues are more relevant to chitinous material recovery and co-substrate strategies than direct high-performance larval growth; and aquaculture sludge is more suitable for nutrient recovery or frass-oriented pathways unless feed-grade safety is demonstrated [8,13,14,21,50,53,56]. Therefore, the value of marine insect bioconversion should be judged by product specifications rather than by larval biomass yield alone.

Optimization of marine insect biorefineries should be treated as a multi-objective process rather than a yield-maximization problem. For insect meals, optimization should include true protein, essential amino acid balance, digestibility, ash, chitin, palatability, and contaminant levels. For insect oil, fatty acid composition, EPA and DHA enrichment, n-3/n-6 ratio, free fatty acids, peroxide value, anisidine value, and oxidative stability should be considered. For hydrolysates, peptide profile, solubility, bitterness, digestibility, and bioactivity should be evaluated. For chitin/chitosan fractions and frass, purity, residual protein or ash, heavy metals, maturity, salinity, and biological safety should be included. Therefore, future process development should optimize both conversion performance and product-specific quality specifications.

### 6.6. Product-Quality Specifications for Marine-Fed Insect Biorefineries

To support practical application, each insect-derived product from marine by-product feeding should be linked to a defined quality-control framework. For insect meal, key parameters include crude protein, true protein, amino acid profile, pepsin or species-specific digestibility, ash, salt, chitin, lipid oxidation, microbial load, and contaminants [18,19,96,97]. For insect oil, the minimum quality profile should include fatty acid composition, especially lauric acid, EPA, DHA, total PUFA, n-3/n-6 ratio, free fatty acids, peroxide value, anisidine value, and oxidative stability [8,21,22,98,99]. For chitin and chitosan, extraction yield, purity, residual protein, residual ash, degree of deacetylation, molecular weight, viscosity, and heavy-metal content are required [23,24,100]. For frass, electrical conductivity, maturity, nitrogen form, nutrient composition, heavy metals, pathogens, and phytotoxicity should be assessed before agricultural use [25,26,56,101].

This quality-specification approach is essential because marine by-products are heterogeneous. Without product-level standards, insect bioconversion risks being evaluated only as waste treatment, which is insufficient for feed, fertilizer, or biomaterial applications. In contrast, when substrate selection, microbial pretreatment, insect species, and processing conditions are matched to product specifications, marine by-products can be converted into standardized insect-derived meals, oils, chitinous materials, and fertilizers. This shifts the field from opportunistic waste feeding toward designed insect-based marine biorefineries. The major insect-derived product streams generated from marine by-product feeding, together with their application potential and product-specific quality-control requirements, are summarized in Table 4.

## 7. Applications in Aquafeed and Other Bioproducts

Insect-derived products generated from marine by-product feeding should be evaluated through both product specifications and final applications. The preceding section defined the main product streams and quality-control parameters for marine-fed insect meal, oil, chitinous fractions, and frass [18,19,23,24,25,26,96,97,98,99,100,101]. This section extends that product-quality framework to practical aquafeed and bioproduct applications. Existing studies show that insect meals and oils can replace or complement conventional protein and lipid ingredients in aquafeeds, whereas more recent trials increasingly evaluate functional endpoints such as intestinal health, immune responses, antioxidant capacity, gut microbiota, and disease resistance [18,19,20,23,96,97,98,99,100,102,103,104,105,106,107,108,109,110]. Therefore, marine by-product-fed insects should be framed not as a single fishmeal substitute, but as application-specific bioproduct platforms that generate aquafeed proteins, lipid ingredients, functional additives, chitin/chitosan materials, hydrolysates, and fertilizer products.

### 7.1. Insect Meal as an Aquafeed Protein Ingredient

Insect meal is the most immediate application of marine by-product-fed insects in aquafeeds. BSFL meal, mealworm meal, lesser mealworm meal, and housefly larvae meal have already been evaluated as alternative or complementary protein sources for fish and crustacean feeds [18,19,57,68,69]. Their value depends on crude and true protein, essential amino acid balance, digestibility, ash content, lipid level, chitin content, and processing method rather than crude protein alone [18,19,96,97]. In Atlantic salmon, BSFL meal has shown high protein digestibility and acceptable amino acid digestibility, supporting its use as a fish feed ingredient [96,97]. These existing data provide the nutritional basis for considering marine-fed insect meal as an aquafeed ingredient.

Recent feeding trials add a more application-oriented layer to this foundation. In Pacific white shrimp, dietary BSFL meal has been evaluated not only for growth performance, but also for intestinal health, gut microbial structure, and resistance to *Vibrio parahaemolyticus* [102]. Nunes et al. further showed that BSFL meal could cost-effectively replace fishmeal in practical nursery diets for post-larval *Penaeus vannamei* under high-density culture when nutrient balance and price sensitivity were considered [104]. In rainbow trout, BSFL meal feeding altered gut microbiota, immune-related gene expression, and resistance to *Lactococcus petauri* [106]. Defatted BSFL meal has also been reported to influence health, muscle texture, and intestinal microbiota in Pacific white shrimp [107]. These studies support the transition from a simple replacement narrative to a formulation-specific and health-oriented aquafeed application.

Marine-fed insect meal should therefore be described as a customizable ingredient rather than a generic fishmeal substitute. Larvae produced on clean fish offal, trimmings, or aquaculture by-products may provide protein-rich biomass with marine-associated nutrients [8,21,50,62]. In contrast, insects produced on shell-rich, seaweed-rich, or sludge-like substrates require a stricter control of ash, salt, iodine, chitin, and contaminants before feed use [14,30,53,56]. The practical value of marine-fed insect meal should be assessed using digestible protein, amino acid balance, palatability, gut health, immune response, safety, and final product quality rather than biomass yield alone [18,19,96,97,102,104,106,107]. Representative aquafeed studies using BSFL meal and other insect-derived meals as fishmeal replacements or alternative protein ingredients are summarized in Table 5.

These examples indicate that insect meals can replace or complement fishmeal in several aquatic species, but their value should not be assessed only by crude protein or biomass yield. Species-specific inclusion levels, amino acid balance, digestibility, chitin content, processing method, palatability, intestinal health, pathogen resistance, and economic feasibility should be considered together when insect meals are developed as aquafeed protein ingredients.

### 7.2. Insect Oil as an Alternative and Functional Lipid Ingredient

Insect oil is a second major application because lipid extraction from full-fat larvae generates both a concentrated protein meal and a separate lipid fraction. Conventional BSFL oil is generally rich in saturated and medium-chain fatty acids, especially lauric acid, which can provide dietary energy and may contribute to antimicrobial or gut-modulating effects [18,19,98]. BSFL oil has been evaluated as an alternative lipid source in gilthead seabream juveniles and as a partial fish oil replacement in juvenile mud crab diets [98,99]. These existing aquafeed studies provide the baseline evidence that insect oil can be incorporated into aquatic animal diets.

More recent studies indicate that insect oil may also contribute to functional feed responses. Kumar et al. reported that BSFL supplementation prevented soybean meal-induced intestinal enteritis in rainbow trout and discussed the health benefits of BSFL oil [109]. Fawole et al. further evaluated BSFL oil as a potential substitute for fish or soy oil in juvenile rainbow trout diets [110]. In Pacific white shrimp, combined inclusion of BSFL meal and oil improved growth-related performance and resistance to *Vibrio harveyi* infection under experimental conditions [105]. These studies support the use of insect oil not only as an energy source, but also as a lipid fraction with potential health-relevant effects [105,109].

Marine by-product feeding adds value because it can tailor insect lipid profiles. Fish offal and fisheries or aquaculture by-products can enrich BSFL biomass with omega-3 fatty acids, whereas seaweed or microalgal residues may contribute EPA, iodine, vitamin E, and other marine-associated nutrients [8,21,53,54,63]. However, standard BSFL oil remains limited in long-chain n-3 PUFA compared with fish oil, and enrichment is highly substrate-dependent [8,21,53,54]. Thus, marine-fed insect oil should be positioned as a tunable lipid ingredient that can complement fish oil or vegetable oils rather than completely replace fish oil in all carnivorous aquafeeds. Quality control should include fatty acid composition, EPA, DHA, total PUFA, n-3/n-6 ratio, free fatty acids, peroxide value, anisidine value, and oxidative stability [8,21,22,98,99]. Representative studies evaluating insect oil, BSFL oil, or marine-fed insect biomass as fish-oil complements or functional lipid ingredients are summarized in Table 6.

Overall, insect oil should be described as a complementary or tunable lipid ingredient rather than a universal fish-oil substitute. Standard BSFL oil is useful as an energy source and may provide medium-chain fatty acids, but marine enrichment through fish offal, aquaculture by-products, seaweed, or microalgal residues is needed when EPA, DHA, total PUFA, or n-3/n-6 balance is the target. Therefore, lipid-oriented optimization should include fatty acid composition, EPA and DHA levels, peroxide value, anisidine value, TBARS, storage stability, and contaminant monitoring.

### 7.3. Functional Aquafeeds: Gut Health, Immunity, and Disease Resistance

The most important application shift is from nutrient replacement to functional aquafeed development. Insect meals can contain chitin, chitosan-like compounds, antimicrobial peptides, bioactive peptides, medium-chain fatty acids, minerals, and microbial metabolites that may influence gut health, mucosal immunity, oxidative status, stress response, and disease resistance [18,19,20,100,101]. Farm-like Atlantic salmon work also supports the idea that BSFL meal may function as more than a protein substitute by modulating immune-related parameters [100]. These existing references remain important because they define the mechanistic basis for functional-feed claims.

Recent application studies provide stronger endpoint-level support. In Pacific white shrimp, dietary BSFL meal affected intestinal health and resistance to *Vibrio parahaemolyticus* [102], and fresh BSFL replacement influenced immune enzyme activities, water quality, and intestinal microbiota [103]. Novriadi et al. also reported improved performance and resistance to *Vibrio harveyi* when BSFL meal and oil were used as alternatives to marine ingredients in Pacific white shrimp diets [105]. In rainbow trout, BSFL meal feeding affected the gut microbiota, immune-related gene expression, and *Lactococcus petauri* resistance [106]. A meta-analysis further indicated that BSFL meal can affect fish immune response and antioxidant capacity, although responses vary with species, inclusion level, insect developmental stage, and processing method [108].

Marine-fed insect products could strengthen this functional-feed concept if substrate design is intentional. Fish-derived substrates may contribute amino acids and marine lipids; seaweed and microalgal residues may contribute pigments, iodine, vitamin E, antioxidants, and residual PUFA; and microbial pretreatment may alter peptide, lipid, and metabolite profiles [8,21,53,54,63]. However, functional claims should not be inferred only from ingredient composition. They require feeding trials that evaluate growth, feed conversion, apparent digestibility, intestinal histology, mucosal immunity, antioxidant enzymes, gut microbiota, stress tolerance, and pathogen-challenge survival [102,103,105,106,107,108,109,110]. Representative examples of bioactive and functional aquafeed applications of insect-derived products in aquatic animals are summarized in Table 7.

These studies show that insect-derived aquafeed ingredients are increasingly being evaluated as functional products rather than only as nutrient replacements. However, functional claims should be supported by endpoint-based feeding trials, including growth, feed conversion, apparent digestibility, intestinal histology, mucosal immunity, antioxidant enzymes, gut microbiota, stress tolerance, and pathogen-challenge survival. Therefore, marine-fed insect products should be optimized for both yield and product quality, including bioactive composition, safety, digestibility, palatability, and application-specific performance.

### 7.4. Chitin, Chitosan, and Hydrolyzed Insect Products as Functional Additives and Biomaterials

Chitinous fractions represent another application route because insect biomass contains exoskeletal chitin that can be recovered from larvae, molts, pupal exuviae, or processing residues [23,24]. In aquafeeds, chitin is a double-edged component. It may contribute to immune stimulation and gut microbial modulation, but excessive chitin can reduce nutrient digestibility or limit inclusion levels [20,101]. Therefore, whole insect meal, de-chitinized insect protein, purified insect chitin, and insect-derived chitosan should be regarded as separate product categories rather than one interchangeable material [23,24,101].

Recent aquaculture-focused studies extend this distinction by positioning chitinase treatment and chitosan-based materials as tools for improving insect-meal digestibility, immune modulation, antimicrobial activity, carrier functions, and water-quality management in aquaculture systems [111,112]. These application-oriented findings complement earlier work on insect chitin extraction and characterization, indicating that chitinous insect fractions should be evaluated not only by yield and purity, but also by target-specific functionality [23,24,101,111,112].

Hydrolyzed insect protein is another promising value-added format. Enzymatic hydrolysis can improve solubility, release peptides, improve palatability, and potentially generate bioactive peptide fractions [15,97]. For marine-fed insects, hydrolysates may be especially useful when whole meal is constrained by high lipid, ash, chitin, or sensory issues. However, hydrolysate applications require product-specific validation, including peptide profile, digestibility, nitrogen solubility, bitterness, microbial safety, and biological activity. Thus, hydrolyzed insect products should be positioned as specialty feed additives rather than simple bulk protein replacements.

### 7.5. Frass and Residual Biomass as Circular Fertilizer Products

Frass is a major co-product of insect bioconversion and should be regarded as part of the product portfolio rather than as a disposal residue. The existing insect-bioconversion literature already positions frass and residual substrates as circular nutrient products that may serve as soil amendments or organic fertilizers when maturity, nutrient composition, microbial safety, and phytotoxicity are controlled [25,26,101]. This is particularly relevant for marine by-product systems because fish residues, seaweed, shell waste, and aquaculture sludge can supply nitrogen, phosphorus, calcium, magnesium, iodine, sulfur, and trace minerals that may increase fertilizer value [12,25,26,50,53].

Recent frass studies strengthen this application argument. Abd Manan et al. reviewed the potential of BSF frass as a sustainable organic fertilizer and summarized recent findings on composition, plant-growth performance, and environmental relevance [113]. Gurung et al. showed that manure-derived BSF frass enhanced chili plant growth and altered rhizosphere bacterial communities [115]. Amorim et al. further discussed insect frass composition and its potential use as an organic fertilizer in circular economies [114]. Together, these studies indicate that BSF frass can function as a circular fertilizer product when composition, maturity, plant-growth response, microbial safety, and environmental risks are properly controlled [25,26,101,113,114,115].

Marine-fed frass also carries substrate-specific risks. Seaweed, seafood residues, and aquaculture sludge may increase electrical conductivity, salt load, iodine, arsenic, cadmium, lead, mercury, pathogens, antimicrobial residues, or microplastics [14,55,56]. Therefore, marine-fed frass should be evaluated using nutrient composition, maturity, electrical conductivity, phytotoxicity, pathogen indicators, heavy metals, and substrate-specific contaminants before agricultural application [25,26,56,101,113,114,115]. Substrates that are not suitable for feed-grade insect meal may still be useful for fertilizer-oriented pathways if the contaminant levels and salt loads are acceptable.

### 7.6. Toward Multi-Output Marine Insect Biorefineries

The most realistic application model is a multi-output marine insect biorefinery. Clean fish offal, trimmings, and seafood-processing residues can be directed toward aquafeed-grade insect meal and oil [8,21,62,96,97,98,99,102,103,104,105,106,107,108,109,110]. Seaweed or microalgal residues can be used at controlled inclusion levels to tailor micronutrient, antioxidant, pigment, or fatty acid profiles, but they require contaminant monitoring [53,54,56,63]. Chitinous shell residues can be integrated through cascade strategies that recover pigments, minerals, proteins, chitin, or chitosan before the insect conversion of remaining organic fractions [13,23,24,30,42,111,112]. Aquaculture sludge and higher-risk residues may be better reserved for nutrient recovery, frass production, or waste-stabilization applications unless feed-grade safety is demonstrated [12,14,25,26,50,113,114,115].

This application-specific approach prevents overclaiming. Not all marine by-products should be converted into aquafeed-grade insect biomass, and not all insect products should be marketed as fishmeal replacements. Some products are better positioned as lipid ingredients, functional additives, hydrolysates, chitin/chitosan materials, fertilizer products, or lower-risk circular bioproducts [18,19,20,21,22,23,24,25,96,97,98,99,100,101,102,103,104,105,106,107,108,109,110,111,112,113,114,115]. The value of marine insect bioconversion therefore depends on matching the substrate type, insect biology, pretreatment, processing, product specification, safety requirement, and final application.

Overall, applications in aquafeed and other bioproducts should be built around product specificity. Marine-fed insect meal can support aquafeed protein replacement and functional feed development; insect oil can provide energy, lauric-acid-rich fractions, and partial marine lipid enrichment; chitin, chitosan, and hydrolysates can support feed additives and biomaterial applications; and frass can recover nutrients as fertilizer when maturity and safety are controlled [8,14,18,19,20,21,22,23,24,25,50,53,56,96,97,98,99,100,101,102,103,104,105,106,107,108,109,110,111,112,113,114,115]. This multi-output strategy directly connects the insect bioconversion of fishery discards, seafood processing waste, and marine by-products with the production of functional aquafeeds and value-added bioproducts.

## 8. Safety, Regulation, and Quality Control

Safety is one of the most important barriers to the practical implementation of insect-mediated marine by-product bioconversion. Marine-derived residues are nutritionally attractive substrates, but they may also contain substrate-specific hazards that are less prominent in conventional plant-based insect diets. These hazards include heavy metals, excessive salt and ash, microplastics, spoilage microorganisms, seafood-associated pathogens, dioxins, polychlorinated biphenyls (PCBs), antimicrobial residues, and other aquaculture- or processing-derived contaminants [14,55,56,116,117,118,119]. Therefore, marine-fed insect products should not be evaluated only by larval growth, biomass yield, or waste reduction, but must be assessed through a feed-chain safety framework that links substrate origin, pretreatment, insect species, rearing conditions, processing, product specification, and final application [116,117].

A central principle is that insect bioconversion can transform organic matter but cannot automatically eliminate inorganic or persistent chemical hazards. Heavy metals, arsenic, dioxins, PCBs, microplastics, and some veterinary drug residues may persist, concentrate, or be transferred from substrates into larvae, frass, or residual biomass [14,56,118,119,120,121,122,123]. Thus, successful larval development on a marine by-product does not necessarily mean that the resulting insect meal, insect oil, chitinous fraction, or frass is suitable for feed-grade or fertilizer-grade use. This distinction is particularly important for aquaculture sludge, seaweed residues, shellfish by-products, and mixed seafood-processing wastes, where contaminant profiles may vary according to production system, harvesting area, season, feed inputs, and processing history [14,55,56].

### 8.1. Chemical Hazards and Contaminant Transfer

Heavy metals and metalloids are among the most critical chemical risks in marine by-product-based insect production. Seaweeds, shellfish residues, aquaculture sludge, and sediment-associated materials may contain arsenic, cadmium, mercury, lead, and other trace elements depending on species, cultivation site, environmental exposure, and processing method [14,55,56]. Seaweed-enriched BSFL systems are especially relevant because seaweeds can accumulate iodine and potentially toxic elements, and BSFL reared on seaweed-containing substrates have been reported to accumulate cadmium, lead, mercury, and arsenic [56]. Similarly, studies on insect safety have shown that heavy metals can be transferred from contaminated substrates into insect biomass, with cadmium and arsenic being particularly important concerns [118,119,120,122]. Therefore, heavy metal monitoring should be regarded as a mandatory quality-control step for marine-fed insect products rather than as an optional endpoint analysis.

Persistent organic pollutants are another important concern. Lipid-rich fish residues, aquaculture sludge, and sediment-associated materials may contain dioxins, dioxin-like PCBs, non-dioxin-like PCBs, polycyclic aromatic hydrocarbons, pesticides, or other hydrophobic contaminants [116,119,124]. These compounds are relevant because marine-fed insect oils may be developed as aquafeed lipid ingredients, and lipid fractions can act as carriers for hydrophobic contaminants. Although data on dioxin and PCB transfer in marine-fed insect systems remain limited, the risk should not be ignored when lipid-rich or sludge-derived substrates are used [119]. For this reason, insect oil derived from fish-processing residues, aquaculture sludge, or mixed seafood waste should be evaluated not only for fatty acid profile and oxidative stability, but also for persistent organic pollutants when feed-grade use is intended.

Antimicrobial residues are particularly relevant to aquaculture sludge and farming-derived residues. Uneaten medicated feed, fecal solids, biofloc material, and sludge from intensive aquaculture systems may contain antimicrobial residues or antimicrobial resistance-associated microbial communities depending on farm management history [14,116,117]. Insect treatment may reduce organic load or modify microbial communities, but it should not be assumed to remove antimicrobial residues or eliminate antimicrobial resistance risks. Therefore, sludge-fed insect biomass should be used for aquafeed only when veterinary drug residues, microbial safety, and contaminant transfer have been specifically validated [14,116]. In many cases, high-risk sludge-derived systems may be more defensible for waste stabilization, nutrient recovery, or controlled frass production than for direct feed-grade insect meal production.

### 8.2. Salt, Ash, Lipid Oxidation, and Physicochemical Safety Constraints

Excessive salt is a marine by-product-specific constraint that affects both insect performance and final product quality. Seafood residues may contain salt from seawater, processing brines, salted products, seaweed tissues, shellfish residues, or aquaculture sludge [14,55,56]. High salinity can reduce BSFL growth, alter biomass composition, and impose osmotic stress during larval rearing [72,73]. Therefore, salinity should be measured before substrate formulation, especially when seaweed residues, salted seafood waste, shellfish-processing residues, or sludge-like materials are used.

Salt is not only a rearing-performance issue. High sodium, chloride, and ash contents in the final insect meal or frass may limit their use in aquafeeds or fertilizers. Aquafeed formulations require controlled mineral balance, and excessive ash or salt can reduce ingredient value even when the crude protein content is high. Therefore, marine-fed insect products should be evaluated by ash, sodium, chloride, calcium, phosphorus, and trace mineral contents, not only by crude protein and lipid levels. Washing, soaking, dilution, or co-feeding can reduce salt pressure, but washing generates saline wastewater and may increase the processing costs. Thus, salinity management should be assessed together with larval performance, environmental burden, water use, effluent generation, and final product specification.

High ash and hard mineralized particles are also important in shell- and bone-rich substrates. Crustacean shells, fish bones, scales, mollusk shells, and aquaculture sludge can increase mineral load and reduce the digestible organic fraction available to larvae [30,41,47]. These materials may also affect substrate texture, larval access, residue separation, and downstream processing. Therefore, shell-rich and bone-rich substrates require grinding, sieving, pretreatment, or controlled inclusion levels before insect feeding [13,15,42]. In quality-control terms, high ash content should be interpreted not simply as a nutritional parameter, but also as an indicator of substrate dilution, digestibility limitation, and possible mineral-associated contaminant risk.

Lipid oxidation is another important quality issue for marine-fed insect products. Fish viscera, liver, trimmings, and other lipid-rich residues can improve fatty acid enrichment in BSFL, but they may also increase the oxidation risk during substrate storage, larval rearing, drying, oil extraction, and product storage [8,21,36]. Marine-derived polyunsaturated fatty acids are particularly susceptible to oxidation, which can reduce feed palatability, generate off-odors, and impair nutritional quality. Therefore, peroxide value, *p*-anisidine value, TBARS, free fatty acids, and antioxidant status should be considered for insect oils or high-fat insect meals derived from marine substrates. Drying temperature, storage atmosphere, packaging, and antioxidant strategies should be optimized to preserve product quality.

### 8.3. Microplastics and Physical Contaminants

Microplastics represent an emerging safety concern for marine by-product bioconversion. Fishery discards, seafood-processing residues, aquaculture sludge, seaweed residues, and coastal biomass may be contaminated with plastic fragments, fibers, films, paint particles, or fragments from fishing gear, aquaculture equipment, packaging, and processing environments [121,123]. Unlike proteins, lipids, or polysaccharides, microplastics are not metabolized into insect biomass. Therefore, insect-mediated treatment should not be considered a microplastic-removal technology unless particle fate is directly measured.

Experimental studies indicate that BSFL can ingest and excrete microplastics, but some particles may remain associated with larvae or gut contents depending on particle size, polymer type, exposure level, and starvation or depuration conditions [123]. Analytical methods for detecting microplastics in reared BSFL are also still developing, including approaches based on polarized light optical microscopy and polymer-specific confirmation methods [121]. These findings indicate that a microplastic safety assessment should be incorporated into marine-fed insect systems, especially when substrates originate from aquaculture sludge, coastal debris-associated biomass, mixed seafood waste, or poorly sorted processing residues.

Practical control should begin before larval feeding. Substrates should be screened for visible plastics, packaging residues, net fragments, metal pieces, shell fragments, and other physical contaminants. Depackaging, sieving, sedimentation, density separation, magnetic separation, and visual inspection can reduce physical hazards, but they cannot guarantee the removal of small microplastics. For feed-grade applications, microplastic monitoring should be regarded as an emerging quality-control parameter, particularly when the final product is intended for aquafeeds where consumer and regulatory scrutiny is high.

### 8.4. Biological Hazards, Pathogens, and Spoilage Control

Marine by-products are highly perishable and can undergo rapid microbial spoilage. Fish viscera, blood, liver, trimming residues, mollusk tissues, crustacean residues, and sludge-like materials contain readily degradable proteins, endogenous enzymes, water-soluble nutrients, and high moisture levels that can support rapid microbial growth [2,3]. Spoilage can lead to ammonia formation, pH shifts, biogenic amine production, odor generation, lipid oxidation, and reduced substrate acceptability [2,3,5]. Therefore, substrate freshness, time-temperature history, storage duration, and pretreatment conditions are critical safety variables.

BSFL and other waste-converting insects may reduce certain microbial populations under specific conditions, and previous studies reported reductions in *Salmonella* spp. and *Escherichia coli* during the BSFL treatment of organic wastes [125,126]. However, pathogen reduction should not be interpreted as sterilization. The microbial outcome depends on substrate type, larval density, temperature, moisture, rearing duration, microbial competition, and post-harvest handling [116,117,125,126]. In addition, larvae themselves can carry substrate-associated microorganisms on their surface or in their gut. Therefore, marine-fed larvae intended for feed use require hygienic harvesting, separation from residues, washing or cleaning when appropriate, and validated killing and drying steps.

Pathogen control should follow a hazard-analysis logic. Incoming substrates should be evaluated for microbial load and risk category. High-risk materials such as sludge, fecal solids, spoiled seafood waste, or mixed residues should not be used for feed-grade insect production unless pretreatment and final product safety are validated. Controlled fermentation, acidification, heat treatment, or rapid processing after collection may reduce spoilage and pathogen pressure, but these steps must be standardized. Final insect meal and oil should be tested for relevant microbial indicators and pathogens according to feed or food safety requirements, including *Salmonella* absence, Enterobacteriaceae, *Escherichia coli*, *Listeria* spp. where applicable, total aerobic counts, yeast and mold counts, moisture, and water activity.

### 8.5. Regulatory Considerations for Marine-Fed Insect Products

Because insect-feed regulations differ substantially among jurisdictions, the European Union framework is discussed here as a representative case study; substrate eligibility and product approval must still be verified under local feed, animal by-product, fertilizer, and food-safety regulations.

The regulatory status of marine-fed insect products depends on jurisdiction, insect species, substrate type, processing method, final product form, and target animal species. In the European Union, processed animal proteins derived from approved insect species have been authorized for use in aquaculture feed under Commission Regulation (EU) 2017/893, and later regulatory changes expanded the use of insect-derived processed animal proteins to additional non-ruminant farmed animals under specified conditions [127,128]. However, the authorization of insect protein does not mean that all substrates are permitted. Substrate eligibility, animal by-product category, feed hygiene, undesirable substances, and feed marketing requirements must be considered together [124,129,130,131].

This distinction is crucial for marine by-products. Clean fish-processing by-products may be more defensible as insect substrates than aquaculture sludge, fecal solids, or materials with uncertain contaminant history. Animal by-product regulations determine which materials can enter feed-related production chains, while feed hygiene regulations require traceability, hazard control, sanitation, and safe processing [129,130]. Regulations on undesirable substances establish maximum levels for contaminants such as arsenic, cadmium, lead, mercury, dioxins, PCBs, pesticides, and other hazardous compounds in animal feed [124]. Importantly, contaminated feed materials cannot simply be diluted to meet the safety requirements; contamination must be controlled at the source and through validated processing [124].

The regulatory strategy should therefore be product-specific. Marine-fed insect meal intended for aquafeed requires stricter substrate control, contaminant monitoring, pathogen testing, and documentation than insect biomass intended for lower-risk industrial applications. Insect oil requires additional attention to lipid oxidation and hydrophobic contaminants. Chitin, chitosan, and hydrolysates require the specification of extraction process, purity, residual protein, ash, microbial quality, and possible allergenicity. Frass requires separate consideration as an organic fertilizer or soil improver, including maturity, salinity, heavy metals, pathogens, and local fertilizer regulations [25,26,114]. Thus, regulation should not be regarded as a single approval question, but as a chain-specific compliance framework.

### 8.6. Quality-Control Framework for Marine Insect Biorefineries

**Figure 4:**
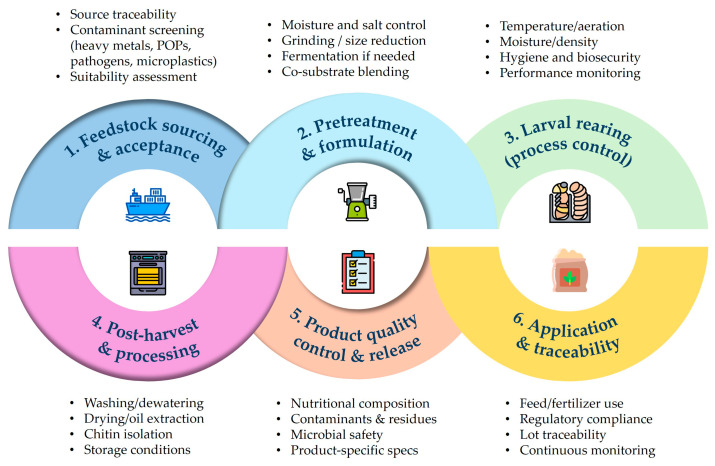
Safety and quality-control gates in marine by-product insect bioconversion chains. The proposed gate-based framework covers six critical stages: feedstock sourcing and acceptance, pretreatment and formulation, larval rearing and process control, post-harvest processing, product quality control and release, and final application with traceability. Each gate should include substrate-specific monitoring points, including contaminant screening, moisture and salt control, particle-size reduction, microbial safety, rearing conditions, post-harvest hygiene, nutritional composition, residue levels, regulatory compliance, and lot traceability. This framework emphasizes that safety should be designed throughout the production chain rather than tested only at the final product stage.

Quality control in marine insect biorefineries should be organized as a series of decision gates from substrate reception to final product release. A gate-based safety and quality-control framework for marine insect biorefineries is proposed in Figure 4. The first gate is substrate acceptance. At this stage, the origin, species, anatomical fraction, processing history, freshness, storage temperature, moisture, salinity, pH, ash content, lipid oxidation status, and visible contamination should be recorded. High-risk substrates should be analyzed for heavy metals, arsenic species where relevant, dioxins, PCBs, pesticides, veterinary drug residues, microplastics, and microbial hazards before they are used for feed-grade insect production [14,55,56,116,117,118,119,120,121,122,123].

The second gate is pretreatment and formulation. Marine substrates may require dewatering, grinding, sieving, washing, fermentation, acidification, heat treatment, or co-substrate blending depending on their dominant limitation [15,22,55,76]. Pretreatment should be validated not only for improved larval growth, but also for safety outcomes, including reduced spoilage, controlled microbial load, manageable salinity, improved texture, and reduced physical contamination. Co-substrate formulation should be documented so that each production lot can be traced back to its input materials.

The third gate is rearing-process monitoring. Larval density, feeding rate, temperature, moisture, pH, substrate depth, aeration, ammonia, odor, mortality, development time, and residue characteristics should be tracked. These variables are not only production parameters; they also indicate whether the rearing system is drifting toward anaerobic spoilage, excessive ammonia formation, microbial instability, or larval stress. Clean and dirty zones should be physically separated, and equipment sanitation should prevent cross-contamination between raw substrates, larvae, processed meals, oils, and frass.

The fourth gate is harvest and processing validation. Larvae should be separated from residues under hygienic conditions and processed using validated killing, drying, defatting, grinding, hydrolysis, or extraction methods. Processing parameters should be selected according to the final product. For whole insect meal, drying must reduce the moisture and water activity sufficiently to prevent microbial growth. For insect oil, extraction and storage should minimize oxidation. For chitin and chitosan, deproteinization, demineralization, deacetylation, and purification steps should be standardized. For frass, maturation, stabilization, salinity, microbial safety, and heavy metal levels should be verified before agricultural use.

The final gate is product release. Marine-fed insect meal should be characterized for proximate composition, amino acid profile, fatty acid profile, ash, minerals, salt, chitin content, digestibility, microbial quality, heavy metals, persistent organic pollutants, antimicrobial residues, microplastics, oxidative stability, and shelf-life. Insect oil should be evaluated for fatty acid composition, free fatty acids, peroxide value, *p*-anisidine value, moisture, impurities, and hydrophobic contaminants. Frass should be evaluated for nutrient composition, maturity, salinity, heavy metals, pathogens, and phytotoxicity. These quality-control endpoints should be aligned with the intended use of each product rather than applied as a generic checklist.

Overall, safety in marine by-product insect bioconversion must be designed into the process rather than tested only at the end. The most defensible strategy is to match substrate risk with product destination. Clean fish trimmings, controlled seafood-processing residues, and validated seaweed or shellfish co-substrates may support aquafeed-grade insect products when the contaminant levels are acceptable. In contrast, aquaculture sludge, highly saline residues, heavily mineralized shell waste, or poorly characterized mixed wastes may be more appropriate for lower-risk nutrient recovery pathways unless rigorous feed-grade validation is performed. This risk-based framework allows insect bioconversion to move from opportunistic waste feeding toward regulated, traceable, and quality-controlled marine biorefineries. To support practical implementation, Table 8 summarizes the major safety, regulatory, and quality-control issues that should be considered when matching marine by-product substrates with feed-, fertilizer-, or bioproduct-oriented insect outputs.

## 9. Sustainability, Techno-Economic Assessment, and Scale-Up

Marine by-product bioconversion by insects is frequently described as a circular and sustainable strategy, but its environmental and economic advantages should not be assumed automatically. Sustainability depends on how the system is designed, what substrate is used, which products are generated, which conventional products are displaced, and how much energy, water, labor, and processing infrastructure are required [132,133,134,135,136]. This point is particularly important for marine by-products because they are often wet, perishable, saline, lipid-rich, heterogeneous, and potentially contaminated. Therefore, marine insect biorefineries should be evaluated not only as waste-reduction systems, but also as integrated resource-recovery systems that include substrate logistics, pretreatment, insect rearing, processing, product use, residue management, and regulatory compliance [14,116,117,134].

### 9.1. Sustainability Potential and Circular Bioeconomy Value

The sustainability value of insect-mediated marine by-product conversion can arise from several mechanisms. First, insects can recover nutrients from low-value or unstable marine residues and convert them into protein-rich biomass, lipid fractions, chitin-containing materials, and frass [5,6,7]. Second, insect-derived meals and oils may partially replace conventional aquafeed ingredients such as fishmeal, fish oil, soybean meal, or vegetable oils, thereby reducing pressure on marine and terrestrial feed resources [18,19,96,98,99]. Third, frass and residual substrates may contribute to nutrient recycling in agricultural systems when safety and maturity criteria are satisfied [25,26,113,114,115]. Fourth, insect bioconversion may reduce the burden associated with conventional disposal routes for wet organic residues, including landfilling, unmanaged decomposition, or low-value disposal [7,132,135,136].

However, the sustainability outcome is highly context-dependent. Marine by-products with high moisture content may require dewatering, drying, or bulking agents before larval feeding, and these operations can increase energy demand and cost [71,74,75,76]. High-salt seaweed or seafood residues may require washing, dilution, or blending, which can generate saline wastewater and additional environmental burdens [55,56,72,73]. Lipid-rich fish residues can improve the value of insect oils, but they may also require cold storage, oxidation control, and careful processing [8,21,36]. Therefore, the environmental benefit of marine-fed insect production should be evaluated through complete life cycle thinking rather than simple claims of waste reduction or protein production [132,133,134,135,136].

### 9.2. Life Cycle Assessment Boundaries, Functional Units, and Environmental Hotspots

Life cycle assessment (LCA) is essential for comparing marine insect bioconversion with alternative waste-management or feed-production pathways. Previous LCA studies on BSF systems have shown that results are strongly influenced by system boundaries, allocation methods, functional units, substitution assumptions, energy use, and avoided waste-treatment credits [133,135,136,137]. In marine by-product systems, these methodological choices become even more important because the process is multifunctional. A single system may simultaneously treat wet waste, produce insect meal, generate insect oil, recover chitinous fractions, and produce frass. Therefore, LCA studies should clearly define whether the functional unit is based on wet waste treated, dry matter processed, digestible protein produced, aquafeed ingredient produced, lipid recovered, nitrogen or phosphorus recycled, or economic value generated.

Several environmental hotspots should be considered. Drying is likely to be a major energy-consuming step because marine substrates and larvae contain high moisture levels [71,74,75]. Refrigerated storage and rapid transport may be necessary for fish viscera, trimmings, and other highly perishable residues. Washing or desalting steps may reduce salt pressure but can increase water use and effluent-treatment requirements. Defatting, oil extraction, grinding, hydrolysis, chitin extraction, and packaging also add processing impacts. In addition, direct emissions from rearing substrates, including CO_2_, CH_4_, N_2_O, NH_3_, and odor-related compounds, should be measured rather than assumed negligible [135,137,138]. Thus, the sustainability of marine insect biorefineries depends on process integration, local energy mix, substrate proximity, valorization of multiple outputs, and the degree to which high-impact conventional ingredients or disposal routes are replaced.

### 9.3. Techno-Economic Assessment and Value Drivers

Techno-economic assessment (TEA) should be used together with LCA because an environmentally promising system may still fail if substrate logistics, processing cost, labor demand, regulatory compliance, or product-market value are unfavorable. For marine insect biorefineries, the major cost drivers include substrate collection, transport, cold storage, dewatering, grinding, salinity management, fermentation or other pretreatments, rearing infrastructure, climate control, labor, automation, drying, defatting, quality testing, packaging, and waste or effluent treatment [15,71,74,75,76,139]. Safety monitoring can also become a substantial cost when feed-grade products are targeted, especially for substrates with potential heavy metals, arsenic, dioxins, PCBs, antimicrobial residues, pathogens, or microplastics [14,56,116,117,118,119,120,121,122,123,124].

Revenue can come from several streams, including tipping fees for waste treatment, insect meal, insect oil, chitinous fractions, hydrolysates, frass, and specialized functional aquafeed ingredients. However, the relative importance of these revenue streams will differ according to substrate quality and product destination. Clean fish-processing residues may support higher-value aquafeed-grade insect meal and oil. Seaweed-containing substrates may generate marine micronutrient-enriched biomass, but only if iodine, arsenic, cadmium, and other contaminants remain within safe limits [53,56]. Crustacean shell residues may be more economically attractive in a cascade biorefinery where pigments, proteins, minerals, or chitin are recovered before or alongside insect treatment [42,44]. Aquaculture sludge may provide waste-management value, but it may be less suitable for high-value feed-grade insect products unless contaminant transfer is rigorously controlled [12,14].

A realistic TEA should therefore avoid treating insect biomass as a single commodity product. Instead, it should evaluate substrate-specific product pathways. For example, one scenario may prioritize waste stabilization and frass production from high-risk sludge, whereas another may prioritize feed-grade insect meal and oil from clean fish trimmings. A third scenario may use seaweed or microalgal residues only as low-inclusion functional co-substrates for nutrient tailoring. This product-specific TEA logic is more appropriate for marine by-products than a generic “waste-to-protein” model.

### 9.4. Scale-Up Bottlenecks and Process Engineering

Scaling marine insect bioconversion from laboratory trials to industrial operation requires the control of both biological and engineering variables. At small scale, larvae may grow successfully on a given substrate, but industrial performance depends on consistent egg supply, larval density, feeding rate, substrate depth, heat accumulation, aeration, moisture, pH, ammonia, odor, rearing time, harvesting efficiency, and residue separation [5,60,71,74,75]. Marine substrates intensify these challenges because they can liquefy, compact, spoil, oxidize, or accumulate salt more rapidly than many plant-based side streams [2,3,14,55,56].

Feedstock standardization is one of the most important scale-up requirements. Industrial systems need predictable substrate specifications, including moisture, dry matter, crude protein, lipid, ash, salinity, particle size, pH, microbial load, and contaminant profile. Without these data, larval performance, product composition, and safety outcomes will vary between production lots. Marine residues should therefore be classified before processing into clean feed-grade substrates, conditional co-substrates, pretreatment-required substrates, and high-risk substrates unsuitable for feed-grade production. This classification can reduce process failure and prevent unsafe materials from entering high-value product chains [14,116,117].

Facility design should also reflect the risk profile of marine by-products. Clean and dirty zones should be physically separated. Raw substrate handling, larval rearing, harvesting, killing, drying, defatting, milling, oil storage, and frass handling should follow hygienic process flow. High-moisture substrates may require dewatering, absorbent co-substrates, forced aeration, shallow bed depth, or modular trays to prevent anaerobic zones [71,74,75,76]. High-salt substrates may require controlled dilution or inclusion limits [72,73]. Lipid-rich substrates may require antioxidant strategies and low-oxygen storage to protect insect oil quality [36]. These engineering decisions directly affect productivity, safety, product consistency, and economic feasibility.

### 9.5. Implementation Pathways for Fisheries and Aquaculture Systems

Marine insect biorefineries are most likely to succeed when they are integrated into existing fisheries, seafood-processing, aquaculture, or seaweed-processing infrastructure. Co-location with fish-processing plants can reduce transport distance, spoilage risk, refrigeration cost, and substrate variability. Aquaculture farms may provide sludge, uneaten feed, fish mortalities, or trimming residues, but these streams should be separated by risk level rather than pooled indiscriminately. Seaweed-processing facilities may provide carbohydrate- and mineral-rich residues, but these should be used at controlled inclusion rates with contaminant monitoring [53,55,56].

A practical implementation model may involve three linked stages. The first stage is substrate triage and pretreatment, where residues are sorted, screened, dewatered, ground, fermented, washed, or blended according to their dominant limitation. The second stage is insect conversion, where optimized larval rearing conditions are maintained for each substrate category. The third stage is product fractionation and quality control, where insect biomass is separated into meal, oil, hydrolysate, chitinous fractions, or other products according to market and safety requirements. This staged model is better suited to marine by-products than direct feeding of mixed residues because it allows each substrate to be matched with a defensible product pathway.

Overall, the feasibility of marine insect bioconversion depends on aligning substrate supply, process design, product value, safety control, and local market demand. Near-term implementation is most defensible for clean fish-processing residues and controlled seafood by-products directed toward aquafeed-grade insect meal and oil. In contrast, high-risk substrates such as aquaculture sludge, highly saline residues, or poorly characterized mixed wastes are better positioned for waste stabilization, nutrient recovery, or frass-oriented applications unless feed-grade safety is rigorously validated.

## 10. Limitations, Future Directions and Conclusions

### 10.1. Limitations of the Current Evidence Base

The available evidence remains uneven across marine substrate classes and insect species. Most direct studies focus on BSFL, whereas yellow mealworms, lesser mealworms, housefly larvae, and other dipterans have much weaker direct evidence for marine by-product conversion. In addition, many studies report larval growth or waste reduction but do not provide complete substrate characterization, contaminant transfer data, digestibility, shelf-life, or product-level quality specifications.

Comparisons across studies are further complicated by differences in substrate dry matter, larval density, feeding rate, rearing duration, inclusion level, pretreatment method, and calculation methods for bioconversion efficiency. Therefore, the framework proposed here should be interpreted as a decision-support model requiring validation through standardized, pilot-scale, product-specific studies rather than as a universal protocol for all marine by-products.

### 10.2. Future Directions and Conclusions

Insect-mediated bioconversion offers a promising route for transforming fishery discards, seafood processing waste, aquaculture residues, crustacean shell waste, seaweed by-products, and other marine side streams into circular aquafeed ingredients and value-added bioproducts. However, the central requirement is substrate-specific design. Marine by-products differ widely in moisture, salinity, lipid content, mineral load, particle structure, spoilage risk, contaminant profile, and product suitability. Therefore, future studies should move beyond simple demonstrations of larval survival or waste reduction and instead evaluate how defined marine substrates can be converted into safe, consistent, and market-relevant insect-derived products.

Several priorities should guide this transition. First, substrate characterization should be standardized across studies, including dry matter, protein, lipid, ash, salinity, pH, mineral profile, microbial load, oxidation status, and contaminant levels. Second, substrate–insect matching should be refined using performance and safety endpoints rather than growth alone. BSFL remain the most defensible platform for wet and nutrient-rich marine residues, whereas mealworms and lesser mealworms are better positioned for dried or formulated marine-derived ingredients. Third, microbiome-assisted strategies, including fermentation, enzymatic pretreatment, and selected microbial inoculation, should be developed to stabilize perishable substrates, improve digestibility, and tailor insect product quality.

Product-specific quality control is equally important. Marine-fed insect meal, oil, hydrolysates, chitinous fractions, and frass should not be regarded as interchangeable outputs. Feed-grade products require the strict validation of heavy metals, arsenic, persistent organic pollutants, antimicrobial residues, pathogens, microplastics, salinity, oxidation, digestibility, and shelf-life. In contrast, high-risk substrates such as aquaculture sludge or poorly characterized mixed residues may be more appropriate for lower-risk nutrient recovery or frass-oriented pathways unless rigorous feed-grade validation is performed. Future pilot-scale studies should therefore integrate LCA, TEA, regulatory feasibility, and product safety from the beginning rather than after process optimization.

In conclusion, marine insect bioconversion should be viewed as a controlled biorefinery strategy rather than a simple waste-feeding approach. Its strongest contribution lies in matching specific marine residues with suitable insect platforms, pretreatment methods, and product destinations. Clean fish-processing residues are the most realistic near-term substrates for aquafeed-grade insect meal and oil; seaweed and microalgal residues may serve as functional co-substrates for nutrient tailoring; crustacean shell residues are better suited to integrated pretreatment and chitin-oriented biorefineries; and aquaculture sludge should be managed cautiously as a high-risk nutrient-recovery substrate. With substrate classification, microbiome-assisted pretreatment, safety validation, process engineering, and product-specific regulation, marine insect biorefineries could become a practical component of sustainable fisheries, aquaculture, and the blue bioeconomy.

## Figures and Tables

**Table 1:** Classification of marine by-products as feedstocks for insect bioconversion.

Feedstock Type	Representative Substrates	Key Constraints for Insect Conversion	Recommended Strategy/Main Output	References
Protein-rich soft tissues	Fish heads, frames, viscera, blood, liver, trimmings	High moisture; rapid spoilage; odor/ammonia; lipid oxidation	BSFL-centered conversion; dewatering/co-feeding/ fermentation; meal and oil	[2,3,8,11,21,29,31,33]
Lipid-rich fish residues	Viscera, liver, roe, fatty trimmings	Rancidity; greasy texture; substrate compaction; variable fatty acid transfer	Controlled BSFL inclusion; dry/fibrous co-substrates; omega-3-tailored biomass/oil	[8,9,10,21,22,36]
Collagenous/ mineralized fractions	Fish skin, scales, bones, swim bladder, mollusk shells, squid pens	Low digestibility; high ash; hard texture; poor accessibility	Fraction separation; grinding; extraction-first cascade; limited insect conversion	[34,35,37,38,47,48,49]
Chitinous crustacean residues	Shrimp/crab shells, exoskeletons, cephalothorax, appendages	Rigid chitin–mineral matrix; high ash; salinity	Grinding/deproteinization/ demineralization; co-substrate BSFL conversion; chitin-oriented biorefinery	[13,30,39,40,41,42,43,44,45,46]
Seaweed residues	Brown, red, and green seaweed processing wastes	Complex polysaccharides; high salt/ash; iodine/ arsenic/heavy metal risk	Low inclusion; microbial/ enzymatic pretreatment; nutrient-tailored biomass/frass	[50,51,52,53,54,55,56]
Aquaculture sludge	Sludge, uneaten feed, fecal solids, biofloc, RAS sludge	Contaminants; pathogens; antimicrobials; microplastics; low/variable digestibility	Dewatering and screening; nutrient recovery/frass first; feed-grade only after validation	[12,14,25,50]

**Table 2:** Comparative characteristics of insect platforms for marine by-product bioconversion.

Insect Platform	Suitable Substrate Type	Strengths	Limitations	References
BSFL, *Hermetia illucens*	Wet/protein-rich residues; fish offal; mixed seafood waste; sludge blends	Broad substrate tolerance; rapid growth; biomass/oil/frass production	Sensitive to excess moisture, salinity, lipid load, spoilage, contaminants	[5,6,7,8,11,12,13,14,21,50,62,63,64,71,72,73,74,75,76]
Yellow mealworm, *Tenebrio molitor*	Dried fish powder; hydrolysates; formulated marine ingredients	Established feed insect; useful for dry ingredient upgrading	Poor fit for raw wet waste; drying/formulation required	[58,65]
Lesser mealworm, *Alphitobius diaperinus*	Dry organic side streams; formulated marine ingredients	Dry-substrate compatibility; circular feed potential	Limited direct marine evidence; unsuitable for raw fish waste	[66,67]
Housefly larvae, *Musca domestica*	Moist nitrogen-rich residues; controlled animal-waste streams	Rapid growth; aquafeed ingredient evidence	Biosecurity, sanitation, containment, public-acceptance concerns	[57,68,69]
Other dipteran larvae	Carrion-like meat/fish residues	Experimental animal-waste reduction potential	Pathogen/myiasis risk; weak regulatory maturity	[70]

**Table 3:** Representative studies on insect bioconversion of marine by-products.

Substrate	Insect/Treatment	Main Outcome	Key Implication	References
Fish offal	BSFL; direct feeding	Increased lipid and omega-3 enrichment	Fish residues can tailor insect oil/biomass	[8]
Fisheries/aquaculture by-products	BSFL; formulated diets	Altered growth, body composition, omega-3 PUFA	Substrate composition controls product quality	[21]
Fish waste + fruit residues	BSFL; co-conversion	Changed conversion efficiency and performance	Fish waste works better as a co-substrate	[64]
Aquaculture sludge	BSFL; fresh sludge	Sludge conversion with contaminant-transfer concern	Growth does not equal feed-grade safety	[12,14]
RAS sludge + fish trimmings + macroalgae	BSFL; blended diets	Protein-rich biomass; altered crude fat	Blending enables practical conversion	[50]
Shrimp carcasses	BSFL; shrimp residue feeding	Feasible shrimp-waste conversion	Shell waste needs process control	[13]
Brown algae media	BSFL; seaweed-enriched diet	EPA/iodine/ vitamin E enrichment; reduced growth at high inclusion	Seaweed is useful only at controlled inclusion	[53]
Seaweed + fish offal	BSFL; fermentation/microwave pretreatment	Improved omega-3 enrichment; performance constraints remained	Pretreatment helps but does not solve all limits	[54]
Seaweed-enriched media	BSFL; increasing seaweed inclusion	Cd, Pb, Hg, As accumulation	Seaweed requires contaminant screening	[56]
Microalgal residues	BSFL; residue incorporation	Residual omega-3 enrichment	Microalgae can be functional enrichment feedstock	[63]
Fish by-products	*T. molitor*; pretreated diets	EPA/DHA enrichment; performance depended on physical form	Mealworms need processed marine ingredients	[65]
Processed housefly larvae meal	Aquafeed ingredient trials	Fishmeal-replacement potential	Feed value shown; waste-conversion role less mature	[68,69]

**Table 4:** Insect-derived products from marine by-product feeding and application potential.

Product	Marine-Feeding Effect	Main Applications	Key QC Points	References
Insect meal	Protein/mineral enrichment; possible ash/salt increase	Aquafeed protein; functional feed ingredient	True protein, amino acids, digestibility, ash, chitin, contaminants	[18,19,21,50,57,68,69,96,97,102,103,104,105,106,107]
Insect oil	Lauric acid base; possible EPA/DHA/PUFA tailoring	Aquafeed lipid; energy source; fish-oil complement	Fatty acids, peroxide value, anisidine value, oxidation, contaminants	[8,21,22,53,54,63,98,99,108,109,110]
Chitin/chitosan fractions	Insect exoskeleton-derived chitin; shell-related mineral issue	Feed additive; immune modulation; biomaterials; carrier systems	Yield, purity, deacetylation, residual ash/protein, heavy metals	[20,23,24,100,101,111,112]
Hydrolyzed insect protein	Peptide-rich fraction; improved solubility/ digestibility potential	Specialty aquafeed additive; palatability/ functional ingredient	Peptide profile, digestibility, bitterness, microbial safety	[15,97]
Frass	Nutrient/mineral recovery; possible salt/metal accumulation	Fertilizer; soil amendment; nutrient recycling	EC, maturity, pathogens, heavy metals, phytotoxicity	[12,14,25,26,50,53,56,101,113,114,115]
Multi-output biorefinery	Product pathway depends on substrate risk	Integrated feed, oil, chitin, frass, biomaterial platform	Product-specific standards and regulatory validation	[8,13,14,21,50,53,56,96,97,98,99,100,101,102,103,104,105,106,107,108,109,110,111,112,113,114,115,116]

**Table 5:** Representative aquafeed studies using insect meals as a fishmeal replacement.

Insect-Derived Ingredient	Target Aquatic Species	Replacement Target	Inclusion/Replacement Level	Main Outcomes	Limitation	References
BSFL meal	Atlantic salmon, *Salmo salar*	Fishmeal	Total fishmeal replacement was evaluated in seawater-phase salmon diets	BSFL meal supported growth, feed utilization, nutrient digestibility, and fillet sensory quality, indicating that it can serve as a major fishmeal alternative in salmon diets	Product quality should be checked by protein digestibility, amino acid digestibility, processing method, and chitin level	[96,97]
BSFL meal	Pacific white shrimp, *LitoPenaeus vannamei*	Fishmeal protein	10, 20, and 30% of fishmeal protein replaced by BSFL meal	Growth was not significantly affected at 10–20% replacement, whereas 30% replacement reduced growth. The 10% replacement group showed improved survival after *Vibrio parahaemolyticus* challenge	Excessive replacement may impair growth. Optimal inclusion should balance growth, intestinal health, and pathogen resistance	[102]
BSFL meal	Post-larval Pacific white shrimp, *Penaeus vannamei*	Fishmeal	Practical nursery diets under high-density culture	BSFL meal could cost-effectively replace fishmeal when nutrient balance and price sensitivity were considered	Economic benefit depends on formulation cost, amino acid balance, and practical nursery conditions	[104]
BSFL meal and oil	Pacific white shrimp, *LitoPenaeus vannamei*	Marine ingredients	Alternative to marine ingredients	Combined BSFL meal and oil improved growth-related performance and resistance to *Vibrio harveyi* infection	Functional effects should be validated under farm-scale and challenge-test conditions	[105]
Defatted BSFL meal	Pacific white shrimp, *Penaeus vannamei*	Fishmeal/protein ingredient	Defatted BSFL meal inclusion	Defatted BSFL meal influenced health, muscle texture, and intestinal microbiota	Defatting improves protein concentration but may alter lipid-mediated functional effects	[107]
BSFL meal	Rainbow trout, *Oncorhynchus mykiss*	Protein ingredient/fishmeal alternative	BSFL meal feeding trial	BSFL meal altered gut microbiota, immune-related gene expression, and resistance to *Lactococcus petauri*	Responses are species- and dose-dependent. Pathogen-challenge outcomes should not be generalized without validation	[106]
Housefly larvae meal after hydrophobic-fraction removal	Red seabream, *Pagrus major*	Fishmeal	Complete substitution of diets containing 70% or 40% fishmeal was tested after processing	Processed housefly larvae meal could replace fishmeal when the hydrophobic fraction was removed	Hydrophobic fractions, including catechol-related compounds, may reduce growth and alter intestinal morphology. Processing is essential	[68]
Housefly maggot meal	Nile tilapia, *Oreochromis niloticus*	Fishmeal	Fishmeal substituted at 0, 90, 180, 270, and 360 g/kg diet	Growth performance, flesh quality, innate immunity, and water environment were evaluated, supporting housefly maggot meal as an aquafeed protein candidate	Suitability depends on replacement level, species, sanitation, and product standardization	[69]

**Table 6:** Representative studies using insect oil or marine-fed insect biomass as fish-oil complements or lipid ingredients.

Lipid Ingredient	Target Species/System	Replacement Target	Fatty Acid-Related Effect	Growth/Health Outcome	Key Limitation	References
BSFL oil	Gilthead seabream, *Sparus aurata* juveniles	Alternative dietary lipid source	BSFL oil provides a lipid source rich in saturated and medium-chain fatty acids	Supported the use of BSFL oil as an alternative lipid source in seabream diets	Standard BSFL oil is not equivalent to fish oil because EPA and DHA are low unless the larvae are marine-enriched	[98]
BSFL oil	Juvenile mud crab, *Scylla paramamosain*	Fish oil	Fish oil was replaced by BSFL oil at 25, 50, 75, and 100%	Replacement below 50% did not significantly reduce growth indices, whereas higher replacement levels reduced growth	Best positioned as partial fish-oil replacement rather than universal complete replacement	[99]
BSFL oil	Juvenile rainbow trout, *Oncorhynchus mykiss*	Fish oil or soy oil	BSFL oil was evaluated as a substitute lipid source	BSFL oil could replace fish or soy oil without negatively affecting growth performance and body composition	n-3 LC-PUFA requirements still need attention in carnivorous fish diets	[110]
BSFL meal and oil	Pacific white shrimp, *LitoPenaeus vannamei*	Marine ingredients	Meal and oil together provided protein and lipid fractions	Improved growth-related performance and resistance to *Vibrio harveyi* infection	Effects need validation across formulations, culture systems, and disease models	[105]
Fish-offal-fed BSFL biomass/oil	BSFL production system	Marine lipid enrichment route	Fish offal increased larval lipid content and enriched larvae with omega-3 fatty acids	Demonstrated that marine by-products can tailor insect biomass composition	This is an insect-production study, not a final aquafeed feeding trial	[8]
Fisheries- and aquaculture-by-product-fed BSFL biomass	BSFL production system	Marine nutrient enrichment route	Marine by-products modulated BSFL growth, body composition, and omega-3 PUFA content	Supports substrate-based tailoring of insect-derived lipid/protein ingredients	Biomass quality depends strongly on by-product type and inclusion level	[21]
Seaweed- or microalgal-residue-fed BSFL biomass	BSFL production system	Functional lipid and micronutrient enrichment	Seaweed and microalgal residues can contribute EPA, iodine, vitamin E, and residual omega-3 fatty acids	Useful for producing marine-enriched insect biomass	High seaweed inclusion can reduce larval performance and may increase iodine, arsenic, or heavy-metal risk	[53,54,56,63]

**Table 7:** Bioactive and functional aquafeed applications of insect-derived products in aquatic animals.

Bioactive Product/Fraction	Putative Functional Component	Target Aquatic Species	Functional Endpoints	Main Finding	Safety/Formulation Consideration	References
BSFL meal	Chitin, antimicrobial peptides, bioactive peptides, minerals, microbial-associated compounds	Atlantic salmon, *Salmo salar*	Immune status under farm-like conditions	BSFL meal was evaluated as a functional feed ingredient and modulated immune-related parameters	Functional claims require farm-scale validation and product standardization	[100]
BSFL meal	Chitin-containing protein meal and bioactive insect components	Pacific white shrimp, *LitoPenaeus vannamei*	Growth, intestinal health, gut microbiota, *Vibrio parahaemolyticus* resistance	Dietary BSFL meal affected intestinal health and disease resistance, with 10% replacement showing favorable challenge-test response	Excessive inclusion may reduce growth. Optimal level should be species- and stage-specific	[102]
Fresh BSFL replacement	Whole-larval nutrients, lipids, microbial-associated factors	Pacific white shrimp, *LitoPenaeus vannamei*	Immune enzymes, water quality, intestinal microbiota	Fresh BSFL replacement improved immune enzyme-related responses, water-quality parameters, and intestinal microbiota	Fresh biomass requires strict microbial safety, storage, and processing control	[103]
BSFL meal and oil	Protein fraction, lauric-acid-rich oil, insect-derived bioactive components	Pacific white shrimp, *LitoPenaeus vannamei*	Growth performance and *Vibrio harveyi* resistance	BSFL meal and oil as alternatives to marine ingredients improved growth-related performance and pathogen resistance	Ingredient combination effects should be separated into protein-, lipid-, and bioactive-fraction effects	[105]
BSFL meal	Chitin-containing meal and microbiota-modulating components	Rainbow trout, *Oncorhynchus mykiss*	Gut microbiota, immune-related gene expression, *Lactococcus petauri* resistance	BSFL meal altered gut microbiota, immune gene expression, and disease resistance	Functional outcomes may depend on basal diet, inclusion level, processing, and pathogen model	[106]
BSFL meal	Insect meal-associated immunomodulatory components	Fish species across studies	Immune response and antioxidant capacity	Meta-analysis indicated that BSFL meal can affect fish immune response and antioxidant capacity	Responses vary by fish species, insect stage, inclusion level, and processing method	[108]
BSFL meal/oil	BSFL protein fraction and BSFL oil	Rainbow trout, *Oncorhynchus mykiss*	Soybean meal-induced enteritis, innate immunity, intestinal histology	BSFL meal supplementation at 8 or 16% prevented soybean meal-induced enteritis and BSFL oil showed immunological benefits	Effects should be interpreted in relation to basal diet composition and inflammatory challenge model	[109]
Insect chitin/chitosan fractions	Chitin, chitosan, chitinase-responsive substrates	Aquaculture species broadly	Growth promotion, immune modulation, antimicrobial activity, carrier utility	Chitin/chitosan fractions are promising functional additives, but effects are dose- and species-dependent	Excessive chitin may reduce digestibility. Chitosan quality requires the control of purity, degree of deacetylation, molecular weight, viscosity, and contaminants	[111,112]
Hydrolyzed insect protein	Peptide-rich fraction with improved solubility and digestibility potential	Aquafeed applications broadly	Palatability, digestibility, peptide bioactivity	Hydrolyzed insect proteins may serve as specialty functional ingredients, especially when peptide profile and digestibility are optimized	Bioactivity should be confirmed by feeding trials, peptide profiling, bitterness assessment, and microbial safety testing	[15,97]

**Table 8:** Safety, regulatory, and quality-control matrix for marine insect bioconversion.

Issue	High-Risk Substrates	Product Risk	Control Strategy	References
Heavy metals/ arsenic	Seaweed; sludge; shellfish residues	Larval/frass accumulation; feed-limit exceedance	Source screening; low inclusion; batch testing	[14,55,56,118,119,120,121,122,123]
Salinity/ high ash	Seaweed; salted seafood; shells; sludge	Poor growth; high-ash meal; high-electrical-conductivity frass	Washing/dilution; co-feeding; inclusion control	[14,30,53,54,55,56,72,73]
Pathogens/ spoilage	Viscera; blood; sludge; raw seaweed	Odor, ammonia, microbial safety risk	Cold chain; rapid processing; fermentation; drying/heat treatment	[2,3,5,12,14,15,55,92,93,94]
Antimicrobial residues/ antimicrobial resistance (AMR)	Aquaculture sludge; medicated feed residues	Regulatory rejection; resistant microbes	Residue screening; avoid contaminated lots; safe starter strains	[14,18,92,93,94]
Persistent organic pollutants/ dioxins/ PCBs	Lipid-rich fish residues; sludge; polluted coastal biomass	Transfer into meal/ oil lipid fractions	Source control; lipid-phase contaminant testing	[14,116,119,124]
Micro- plastics	Sludge; coastal waste; mixed seafood residues	Physical contamination; regulatory concern	Source sorting; sludge caution; polymer identification by FTIR/Raman	[14,118,119,120,121,122,123]
Lipid oxidation	Viscera; liver; fatty trimmings; insect oil	Rancidity; reduced palatability; unstable oil	Rapid processing; antioxidant strategy; oxygen/light control	[8,21,22,36,98,99]
Batch heterogeneity	Mixed seafood waste; seasonal residues	Variable growth and product composition	Feedstock classification; blending recipes; acceptance criteria	[2,3,5,15,27,28,29,30]
Frass maturity/ phytotoxicity	Seaweed-, shell-, sludge- containing residues	Salt stress; immature fertilizer; pathogen risk	Composting; EC/maturity testing; contaminant monitoring	[25,26,56,101,113,114,115]
Regulatory acceptability	Animal-derived waste; sludge; unknown mixed residues	Feed approval barrier; market restriction	Separate feed-grade and non-feed pathways	[14,18,25,26,50,56,116,117]

## Data Availability

No new data were created or analyzed in this study. Data sharing is not applicable to this article.

## Abbreviations

The following abbreviations are used in this manuscript:

BSF	Black soldier fly
BSFL	Black soldier fly larvae
DHA	Docosahexaenoic acid
EPA	Eicosapentaenoic acid
LAB	Lactic acid bacteria
LCA	Life cycle assessment
PCB(s)	Polychlorinated biphenyl(s)
PUFA	Polyunsaturated fatty acid(s)
RAS	Recirculating aquaculture system
TBARS	Thiobarbituric acid-reactive substances
TEA	Techno-economic assessment
n-3	Omega-3 fatty acid
n-6	Omega-6 fatty acid
